# Microtubule retrograde flow retains neuronal polarization in a fluctuating state

**DOI:** 10.1126/sciadv.abo2336

**Published:** 2022-11-04

**Authors:** Max Schelski, Frank Bradke

**Affiliations:** ^1^ Axon Growth and Regeneration Group, German Center for Neurodegenerative Diseases (DZNE), Venusberg-Campus 1, Building 99, 53127 Bonn, Germany.; ^2^ International Max Planck Research School for Brain and Behavior, University of Bonn, Bonn, Germany.

## Abstract

In developing vertebrate neurons, a neurite is formed by more than a hundred microtubules. While individual microtubules are dynamic, the microtubule array has been regarded as stationary. Using live-cell imaging of neurons in culture or in brain slices, combined with photoconversion techniques and pharmacological manipulations, we uncovered that the microtubule array flows retrogradely within neurites to the soma. This flow drives cycles of microtubule density, a hallmark of the fluctuating state before axon formation, thereby inhibiting neurite growth. The motor protein dynein fuels this process. Shortly after axon formation, microtubule retrograde flow slows down in the axon, reducing microtubule density cycles and enabling axon extension. Thus, keeping neurites short is an active process. Microtubule retrograde flow is a previously unknown type of cytoskeletal dynamics, which changes the hitherto axon-centric view of neuronal polarization.

## Teaser

The microtubule array in neurites moves retrogradely toward the soma to keep neurites short, which inhibits axon formation.

## INTRODUCTION

Microtubules build the morphology of eukaryotic cells. They support the complex morphology of neurons by forming arrays, composed of more than 100 microtubules each (*1*). These parallel tracks transport cargo within neuronal projections, the neurites (*2*). Within the microtubule array, single microtubules are dynamic; they can be transported (*3*), generated (*4*, *5*), as well as polymerize and depolymerize (*6*). The microtubular array in the neurite itself, however, has been regarded as a stationary structure, even during neuronal development (*7*, *8*). We challenge this view by showing that the microtubule array in developing neurites flows retrogradely from the distal neurite tip to the cell body.

This discovery of microtubule retrograde flow (MT-RF) changes our view on cytoskeletal dynamics in neurons. We demonstrate that this novel view transforms our understanding of neuronal polarization—the process of how neurons single out one neurite to become the axon, whereas the other neurites later become dendrites (*9*, *10*). Formerly, it was thought that enrichment of specific signaling components distinguishes a rapidly growing axon from stationary neurites (*10*–*12*). Our data instead reveal that keeping neurites short is an active process, executed by MT-RF. In the axon, MT-RF is slowed down, enabling robust extension of the microtubule array and, thereby, neuronal polarization. Thus, the newly discovered type of cytoskeletal dynamics controls basic neuronal function. MT-RF might not only be specific to neurons but also influence microtubule-filled processes of non-neuronal cells.

## RESULTS

### The microtubule array in neurites flows retrogradely

**Fig. 1.**
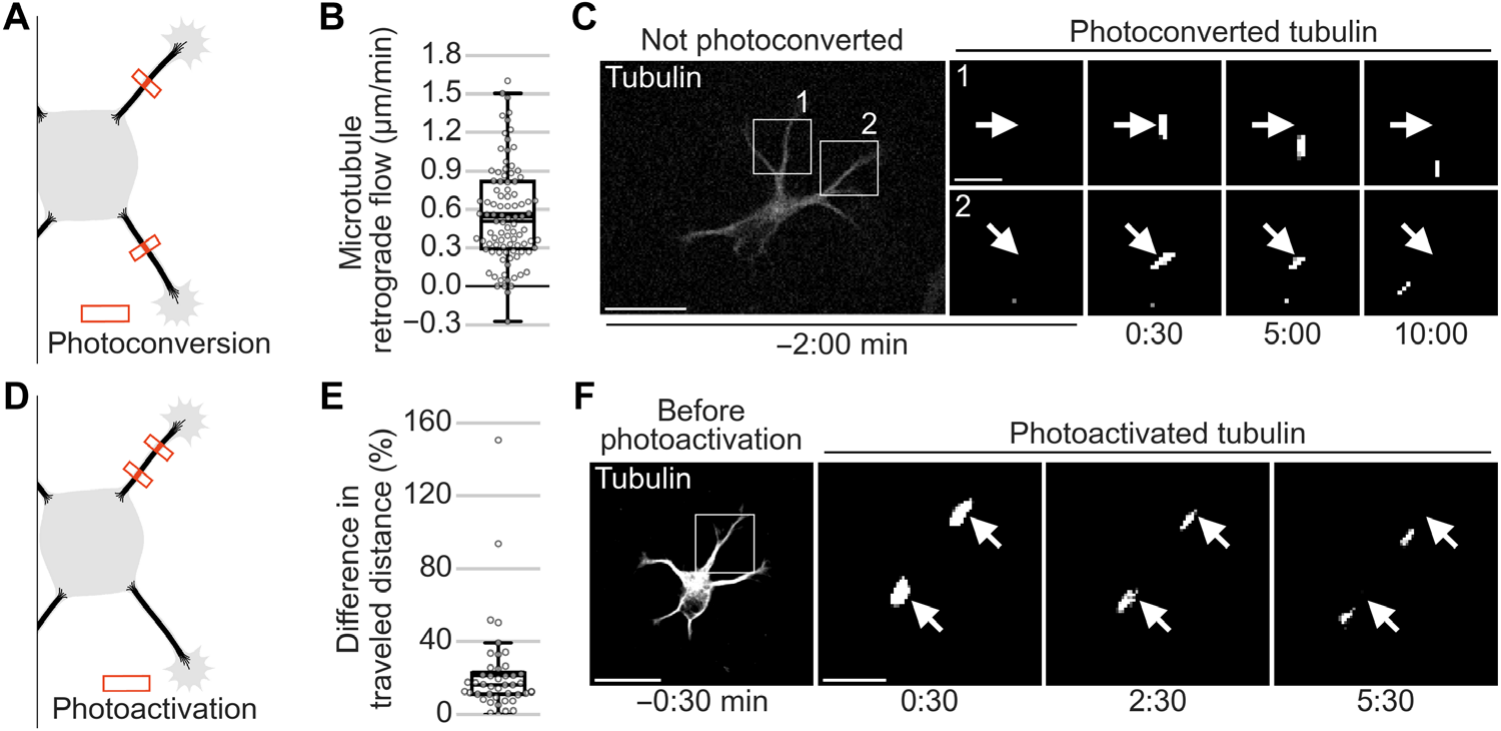
The entire microtubule array in the neurite flows retrogradely before axon growth. The tubulin subtype TUBB2a was fused either (**A** to **C**) to the photoconvertible fluorophore mEos3.2 or (**D** to **F**) to the photoactivatable fluorophore Dronpa expressed in neurons and imaged after 1 day in culture. (A) Illustration of photoconversion experiment for (B) and (C). (B and C) Small microtubule patches were photoconverted in two neurites of neurons without an axon. (B) MT-RF in neurons without axons (*n* = 71 cells, *N* = 11 independent experiments). Each data point represents one neurite. (C) Representative cell for (B). (D) Illustration of photoactivation experiment for (E) and (F). (E) Two or three microtubule patches were photoactivated in a single neurite. For each neurite, the distance difference of the patch that moved furthest and the patch that moved the least was divided by the lower distance (*n* = 44 cells, *N* = 9 independent experiments). (F) Representative cell for (E). The thick line in boxplots shows mean. Scale bars, 20 μm (overview images) and 5 μm (zoomed images).

To visualize the dynamics of the microtubule array in neurites of developing murine hippocampal neurons, we expressed a microtubule subunit (tubulin) fused to the fluorophore mEos3.2 (*13*), which can be photoconverted with ultraviolet light from green to red fluorescence. Through laser-induced local photoconversion of the fluorophore in neurites, we labeled small microtubule patches throughout the whole microtubule stack (fig. S1, A and B) and followed the patches (Fig. 1A) before axon formation. In contrast to what is expected from a stationary microtubule array, the patches quickly moved retrogradely within neurites toward the soma, at speeds of 0.53 ± 0.03 μm/min (mean ± SEM; Fig. 1, B and C, and movie S1). Immobile and anterogradely moving patches were only observed in 4.7 and 1.8% of neurites, respectively (fig. S2, A and B). The speed of MT-RF is slower than microtubule polymerization (12 to 18 μm/min) (*14*, *15*) and fast axonal transport (70 to 280 μm/min) (*16*) but in the range of slow axonal transport (0.2 to 5.5 μm/min) (*8*). MT-RF also occurred in the absence of neurite retraction (fig. S2C). To test whether the entire microtubule array in the neurite moved retrogradely, we followed multiple microtubule patches in the same neurite. To avoid a light-induced stress reaction in the neurite, tubulin was fused to the photoactivatable fluorophore Dronpa (*17*), which needs less light intensity than mEos3.2 to be photoactivated (Fig. 1D). The photoactivated microtubule patches moved retrogradely in a synchronous manner, maintaining a similar distance between each other (Fig. 1, E and F, and movie S2). Accordingly, the speed of retrograde flow of distal patches was, on average, only 12.5% faster than proximal patches (fig. S3, A and B). The actin cytoskeleton appears to be involved in keeping microtubules connected in an array. Photoactivated microtubule patches dispersed both retrogradely and anterogradely more quickly when cells were treated with the actin-depolymerizing drug latrunculin A (LatA; fig. S4, A to D) (*18*). Together, before axon formation, the microtubule array in a neurite flows retrogradely toward the cell body.

### The retrograde flow of the microtubular array is slow in the axon

**Fig. 2.**
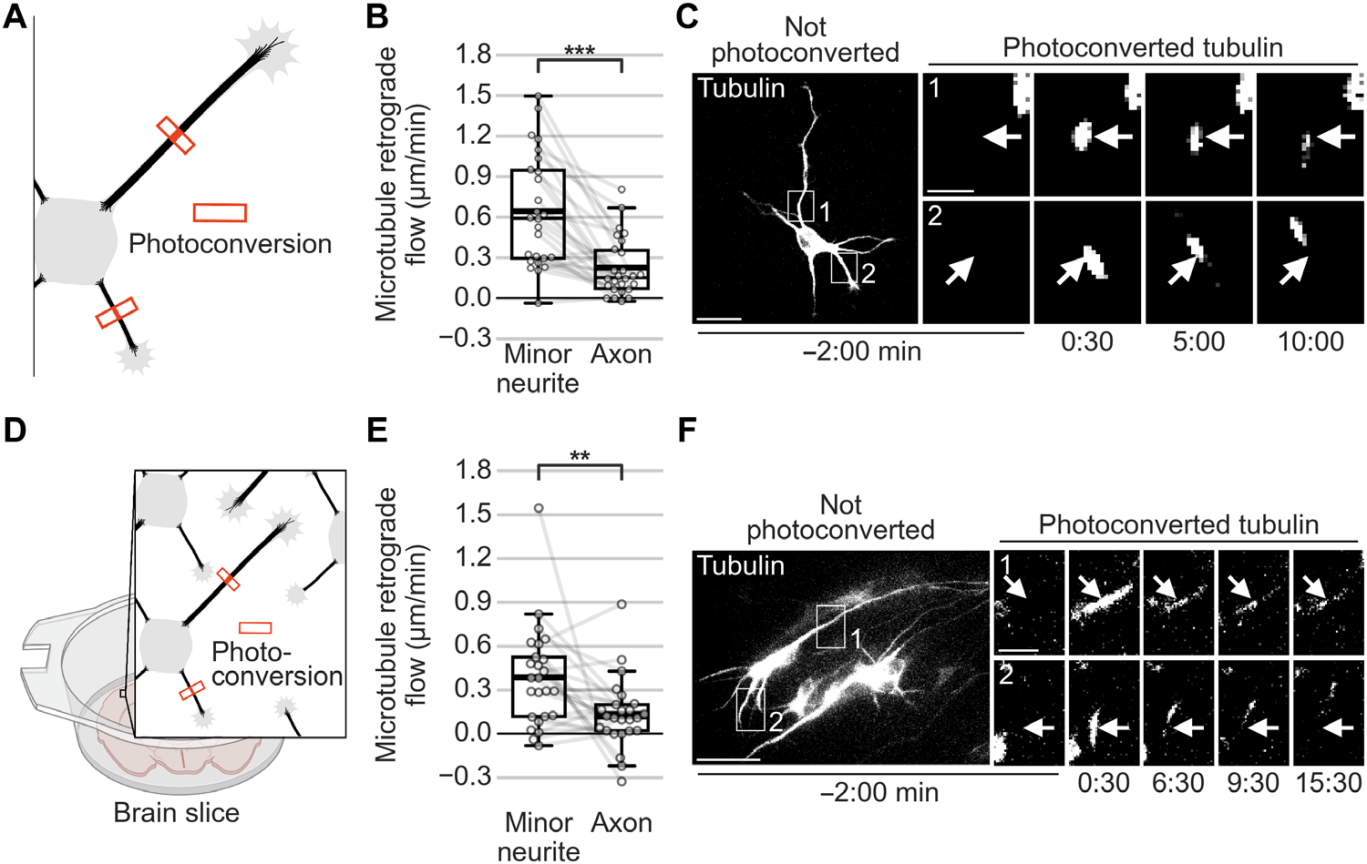
MT-RF slows down in the axon. The tubulin subtype TUBB2a was fused to the photoconvertible fluorophore mEos3.2 expressed in neurons and imaged after 1 day in culture. (**A**) Illustration of photoconversion experiment for (B) and (C). (**B** and **C**) MT-RF in minor neurites and axons (*n* = 26 cells, *N* = 8 independent experiments). Arrows indicate location of photoconversion at 0:00 min. (**D** to **F**) Embryonic brains were electroporated ex utero at embryonic day 14.5 (E14.5) with tubulin subtype TUBB2a, fused to three times photoconvertible fluorophore mEos3.2. Brains were then sliced coronally, kept in the incubator for 2 days, and then imaged. (D) Illustration of the photoconversion experiment for (E) and (F). (E) MT-RF of neurons in brain slices. (*n* = 25 cells, *N* = 8 independent experiments). (F) Maximum intensity projection of the neuron photoconverted at 0:00 min, with arrows pointing to the photoconversion site. The thick line in boxplots shows mean. Arrows indicate the location of photoconversion at 0:00 min. **P < 0.01 and ***P < 0.001, Wilcoxon signed-rank test. Scale bars, 20 μm (overview images) and 5 μm (zoomed images).

Microtubules polymerize into the neurite tip to extend the neurite (*19*), fueled by tubulin that both diffuses (*20*) and is transported (*21*) from the cell body. As neurites remain short before neuronal polarization and after axon formation (*10*, *22*), it raised the possibility that MT-RF might restrain neurite growth by reducing the net extension of the microtubule array. Indeed, MT-RF could be an important hurdle for neurite extension, because MT-RF is 6 to 15 times faster than neurite or axon growth (*5*, *9*, *23*). Consequently, during axon outgrowth, MT-RF may slow down in the axon to enable accelerated growth. Photoconversion of microtubule patches in minor neurites of axon-bearing neurons revealed that MT-RF was similar compared to the neurites of neurons without an axon. However, in the axon, MT-RF was more than twofold slower (0.61 ± 0.08 μm/min in minor neurites compared to 0.23 ± 0.05 μm/min in the axon; mean ± SEM; Fig. 2, A to C, and movie S3). The flow was similar across the entire axon length, as two photoconverted microtubule patches far away from each other moved at similar speeds (fig. S5, A to C, and movie S4). In addition, axons of neurons cultured in three dimensions (3D) (*24*) and of neurons in embryonic organotypic brain slices showed slower MT-RF than minor neurites (Fig. 2, D to F; fig. S6, A to C; and movies S5 and S6). Notably, at later developmental stages, when neurites grow as dendrites (*9*), dendritic MT-RF speed decreased, but not to the level of that found in the axon (fig. S7, A and B, and movie S7).

**Fig. 3.**
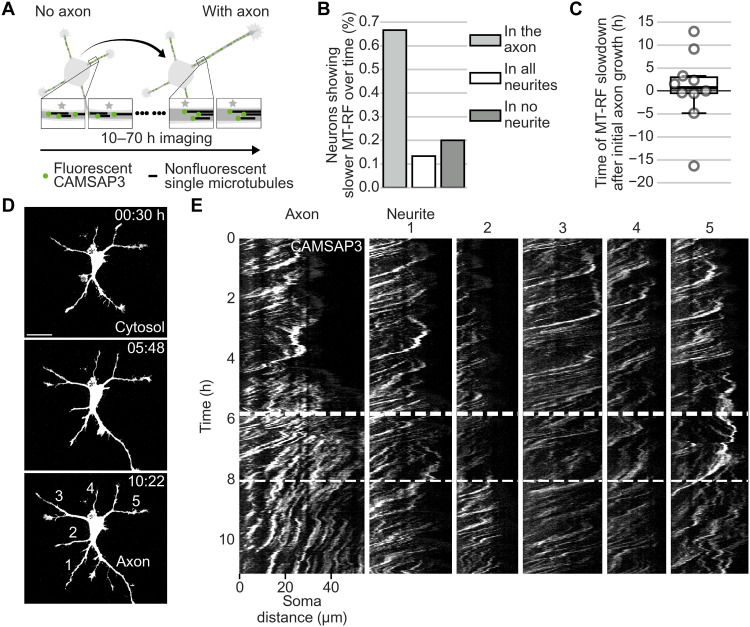
MT-RF slows down shortly after axon growth. CAMSAP3 was fused to the fluorophore mNeonGreen and the cytosolic fluorophore tandem-mCherry (cytosol) expressed in neurons and imaged after 1 day in culture. (**A**) Illustration of long-term imaging of MT-RF by imaging the microtubule (minus) end-binding protein CAMSAP3 for (B) and (C). (**B** and **C**) MT-RF was averaged over 200 min and was considered slowed down once it was 20% slower in the axon compared to all other neurites for 95% of time points for at least 180 min (*n* = 15 cells, from those, 10 cells for time of MT-RF slowdown, *N* = 6 independent experiments). (**D**) Cell morphology of neuron from (E) with the fluorophore tandem-mCherry to label the cytosol before (0:30 hours) and after (5:48 hours) the axon reached axon-like length and when MT-RF slowed down in the axon (10:22 hours). Neurites from (E) are labeled at 10:22 hours. (**E**) Neon-CAMSAP3 intensity along each neurite (*x* axis) over time (*y* axis; kymograph). The left site is close to the soma. Thus, a trace from top right to lower left indicates MT-RF. The thick and thin dashed lines indicate the time when the future axon reached axon-like length (5:48 hours) and when MT-RF slowed down in the axon compared to all other neurites (8:02 hours), respectively. The thick line in boxplots shows mean. Scale bar, 20 μm.

We then defined when MT-RF slowed down in relation to axon formation. As repeated photoconversions are toxic to neurons, we measured MT-RF during neuronal polarization in all neurites simultaneously by expressing and imaging the microtubule minus-end marker calmodulin-regulated spectrin-associated protein 3 (CAMSAP3) (*25*) fused to a fluorophore (Fig. 3A) (*14*). MT-RF slowed down in the axon compared to all other neurites in 66.7% of neurons (Fig. 3B). In these neurons, MT-RF decreased relative to all other neurites approximately 40 min after the initial axon growth (Fig. 3, C to E, and movie S8). Thus, the slowdown of MT-RF in the growing axon could enable its steady growth.

### The retrograde flow of the microtubule array keeps neurons in a fluctuating state

**Fig. 4.**
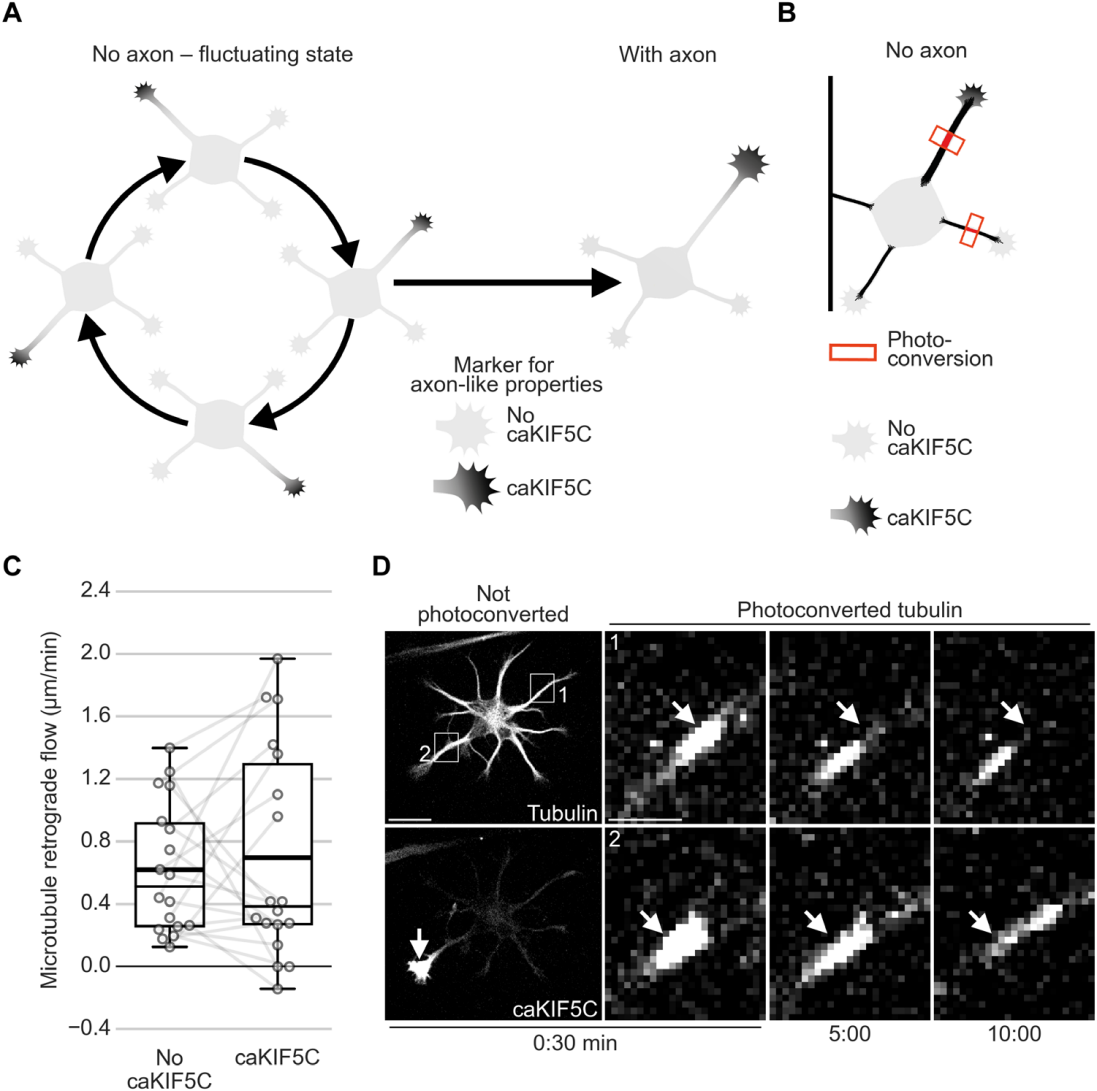
MT-RF does not slow down in neurites with axon-like properties. Neurons expressing the tubulin subtype TUBB2a fused to the photoconvertible fluorophore mEos3.2, and caKIF5C fused to the fluorophore Cerulean3, were cultured for 1 day and then imaged. (**A**) Illustration of fluctuations of caKIF5C before axon formation. (**B**) Illustration of photoconversion experiments for (C) and (D). (**C** and **D**) MT-RF quantified in neurites without and with caKIF5C accumulation in neurons without axons (*n* = 18 cells, *N* = 8 independent experiments). White arrows in the photoconverted channel of (D) indicate areas of photoconversion and, in the caKIF5C channel, point to the growth cone with caKIF5C accumulation. The thick line in boxplots shows mean. *P* > 0.05, Wilcoxon signed-rank test. Scale bars, 20 μm (overview images) and 5 μm (zoomed images).

Before axon formation, neurites fluctuate by transiently acquiring axon-like properties (Fig. 4A) (*10*, *26*, *27*). MT-RF could be the basis of the fluctuating state by continuously acting on all neurites and thereby resetting the repeatedly acquired axon-like state, making this state transient. To label axon-like neurites, we used the constitutively active motor domain of kinesin 1 family proteins (caKIF5C), a key marker for axon-like properties that labels neurite tips and, after axon formation, persistently labels the tip of the axon (Fig. 4A) (*26*). Before axon formation, MT-RF was indistinguishable in neurites that accumulate caKIF5C compared to neurites that did not accumulate this marker (Fig. 4, B to D, and movie S9). In contrast, MT-RF decreased at the transition of axon formation in neurites with caKIF5C accumulation (fig. S8, A to C). Thus, persistent MT-RF slowdown is specific to the axon and does not occur in neurites with axon-like properties. This suggests that before axon formation, MT-RF continuously acts on neurites and could keep neurons in an unpolarized, fluctuating state.

**Fig. 5.**
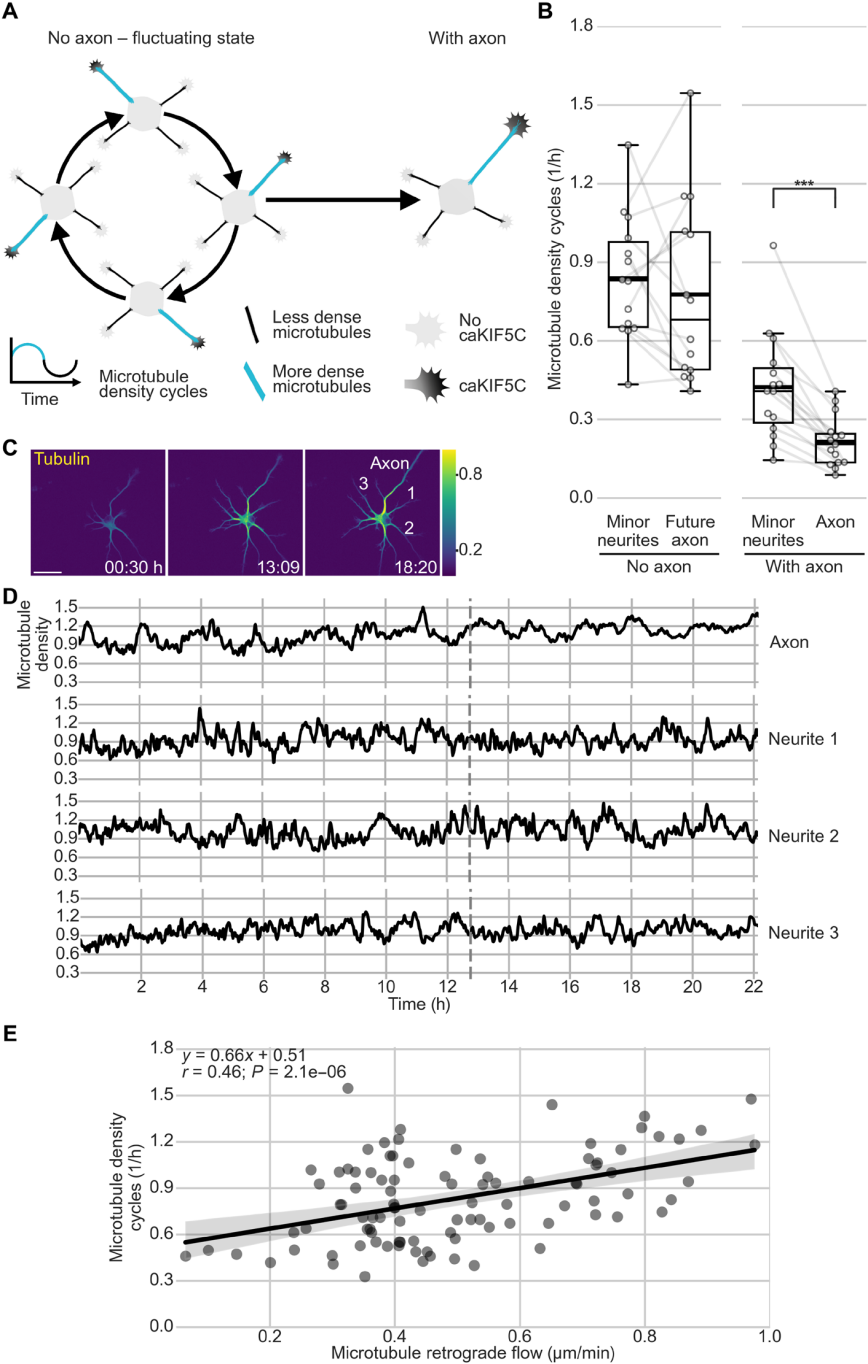
MT-RF could keep neurons in the fluctuating state. Neurons expressing the fluorophore mNeonGreen fused to CAMSAP3 and TUBB2a fused to the fluorophore mScarlet were cultured for 1 day. Then, microtubules and MT-RF were imaged simultaneously in neurons from before to after axon formation. Microtubule density was calculated by dividing the average tubulin intensity by the tubulin expression level in the neuron, modeled as a quadratic function of the average intensity in all neurites for each time point. Changes of average microtubule density in a neurite of 0.2 or more were considered half a cycle. For a full cycle, an increase followed by a decrease, or vice versa, was required [*n* = 15 cells after axon growth (With axon), of those *n* = 14 cells before axon growth (No axon), *N* = 3 independent experiments]. (**A**) Illustration of fluctuations of microtubule density and caKIF5C before axon formation. (**B**) Microtubule density cycles in neurons before and after axon formation. Microtubule density cycles were averaged for all minor neurites of one neuron. (**C** and **D**) Representative cell for (B). (C) Morphology and color-coded tubulin intensity (blue, lower intensity; yellow, higher intensity) at the start of imaging (0:30 hours), directly after (13:09 hours), and some hours after (18:20 hours) axon growth. Neurite numbers from (D) are annotated at the last time frame. (D) Microtubule density was smoothed with a rolling window of 2. The dashed line indicates the time of axon formation. (**E**) Correlation and linear regression of MT-RF with microtubule density cycles (Pearson *r* = 0.46, *P* = 0.0000021; *n* = 95 neurites from 14 cells, *N* = 3 independent experiments). The thick line in boxplots shows mean. ***P < 0.001, Wilcoxon signed-rank test. Scale bar, 20 μm.

During fluctuations, microtubules cycle between low and high density in different neurites (Fig. 5A). While higher density is caused by transient increases in microtubule polymerization (*27*), using the supply of tubulin diffusing and transported from the cell body, the mechanism underlying the decrease of microtubule density has remained elusive. We hypothesized that MT-RF could continuously move microtubules out of the neurite, reducing repeatedly increased microtubule density and thereby fueling microtubule density cycles. Consistent with this hypothesis, axons showed two times fewer microtubule density cycles than minor neurites (Fig. 5, B to D; fig. S9, A to C; and movie S10). In addition, we found an increase in microtubule density cycles in neurites with faster MT-RF before axon formation (Pearson *r* = 0.46; Fig. 5E and fig. S10, A to E).

**Fig. 6.**
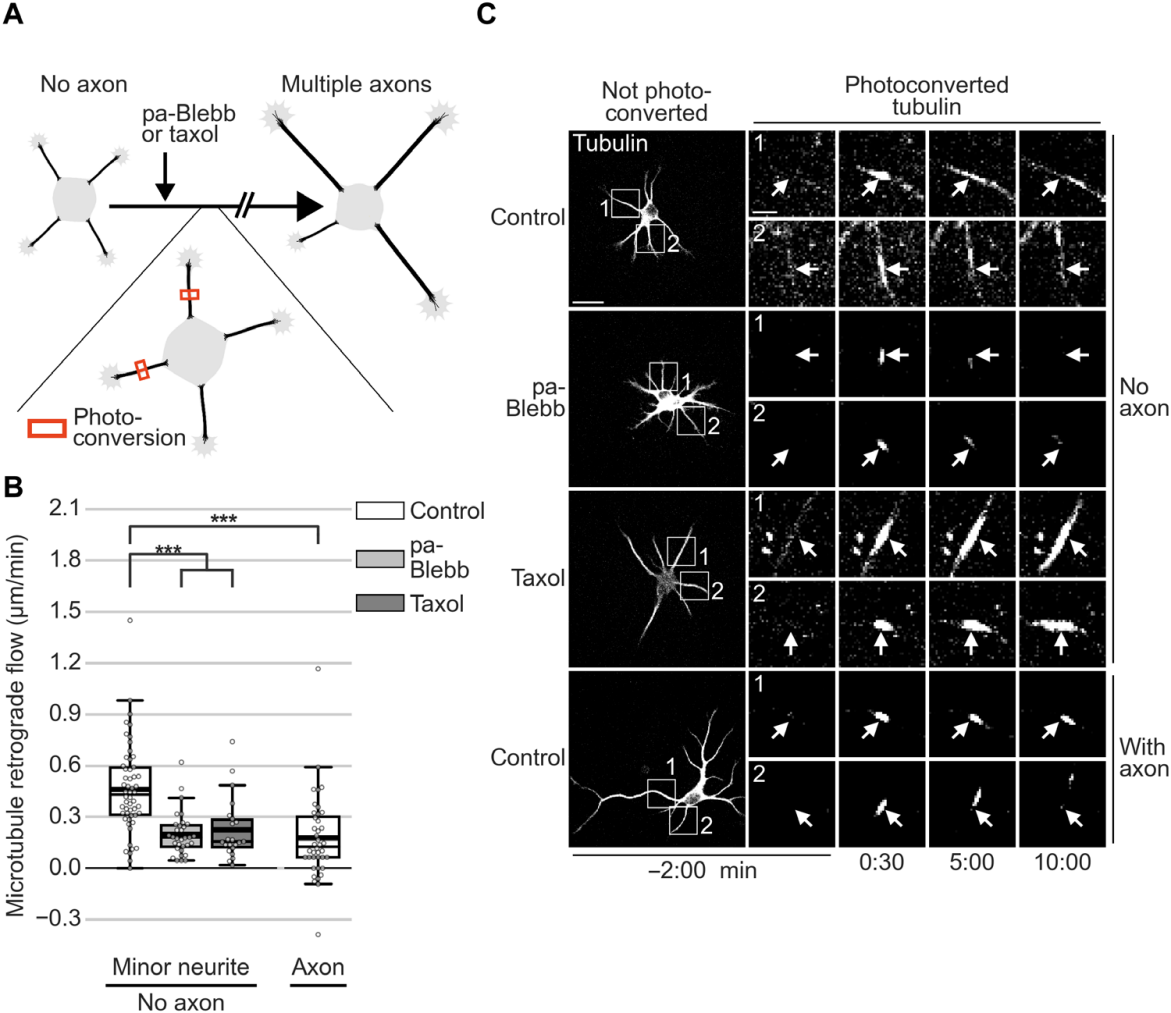
Stabilizing axon identity in multiple neurites slows down MT-RF. The tubulin subtype TUBB2a fused to the photoconvertible fluorophore mEos3.2 was expressed in neurons and imaged after 1 day in culture directly after treating neurons for 20 to 240 min with pa-Blebb (40 μM), taxol (6 nM), or control [dimethyl sulfoxide (DMSO)]. (**A**) Illustration of photoconversion experiment after drug treatments shown in (B) and (C). (**B** and **C**) MT-RF after pa-Blebb, taxol, and control treatment (*n* = 22 cells, *N* = 4 for taxol; *n* = 32 cells, *N* = 4 for pa-Blebb; *n* = 54 cells, *N* = 11 for control without axon; and *n* = 40 cells, *N* = 9 for control with axon; *N*, number of independent experiments). (C) Arrows indicate location of photoconversion at 0:00 min. ***P < 0.001, Kruskal-Wallis multiple comparison with Dunn’s post hoc test with Bonferroni correction. Scale bars, 20 μm (overview images) and 5 μm (zoomed images).

**Fig. 7.**
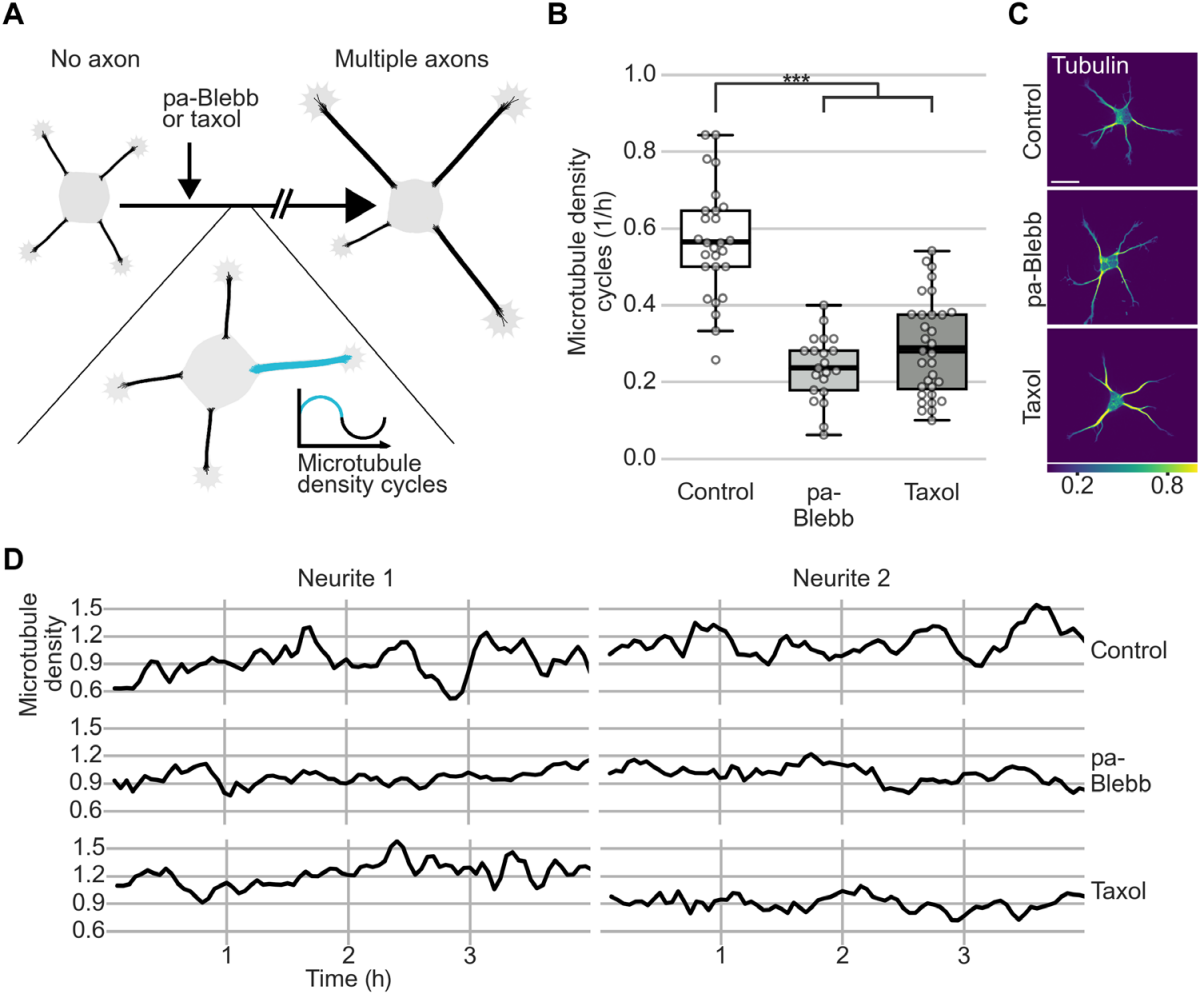
Stabilizing axon identity in multiple neurites reduces microtubule density cycles. The tubulin subtype TUBB2a fused to the fluorophore mNeonGreen was expressed in neurons and imaged after 1 day in culture directly after treating neurons for 20 to 240 min with pa-Blebb (40 μM), taxol (6 nM), or control (DMSO). (**A**) Illustration of experiment after drug treatments shown in (B) and (C). (**B** to **D**) Microtubule density was obtained by normalizing the average tubulin intensity by the tubulin expression level in the neuron, modeled as a linear function of the average intensity in all neurites for each time point. Microtubule density cycles were measured by counting the density increases followed by decreases in neurites of at least 0.2. Neurons were traced, and average intensity along neurites was measured using a self-made fully automated algorithm. (B) The number of cycles per hour was averaged for all neurites of a neuron before axon formation (*n* = 34 cells for taxol, *N* = 3; *n* = 21 cells for pa-Blebb, *N* = 2; and *n* = 27 cells for control, *N* = 3). (C) Morphology and color-coded tubulin intensity (blue, lower intensity; yellow, higher intensity) of neurons at the start of imaging. (D) Microtubule density was smoothed with a rolling window of two frames. The thick line in boxplots shows mean. ***P < 0.001, Kruskal-Wallis multiple comparison with Dunn’s post hoc test with Bonferroni correction. Scale bar, 20 μm.

This raised the possibility that fast MT-RF could prevent neurites from becoming axons by continuously counteracting microtubule accumulation. Consistent with this view, two pharmacological manipulations that transform nongrowing neurites into multiple growing axons per cell (*23*, *28*) slowed down MT-RF in neurites. Even before axon growth, both para-amino blebbistatin (pa-Blebb) and taxol decreased MT-RF to the level of control-treated axons (Fig. 6, A to C, and movie S11). We therefore tested whether these manipulations, in addition to slowing down MT-RF, also decreased microtubule density cycles. Indeed, pa-Blebb and taxol reduced the number of microtubule density cycles by approximately 50% (0.56 ± 0.03 cycles/hour for control, 0.24 ± 0.02 cycles/hour for pa-Blebb, and 0.29 ± 0.02 cycles/hour for taxol; mean ± SEM; Fig. 7, A to D, and movie S12). Thus, slower MT-RF could reduce microtubule density cycles and enable neurites to become axons by hindering the retrograde removal of microtubules from neurites.

### Dynein recruitment to the plasma membrane causes speedup of MT-RF

**Fig. 8.**
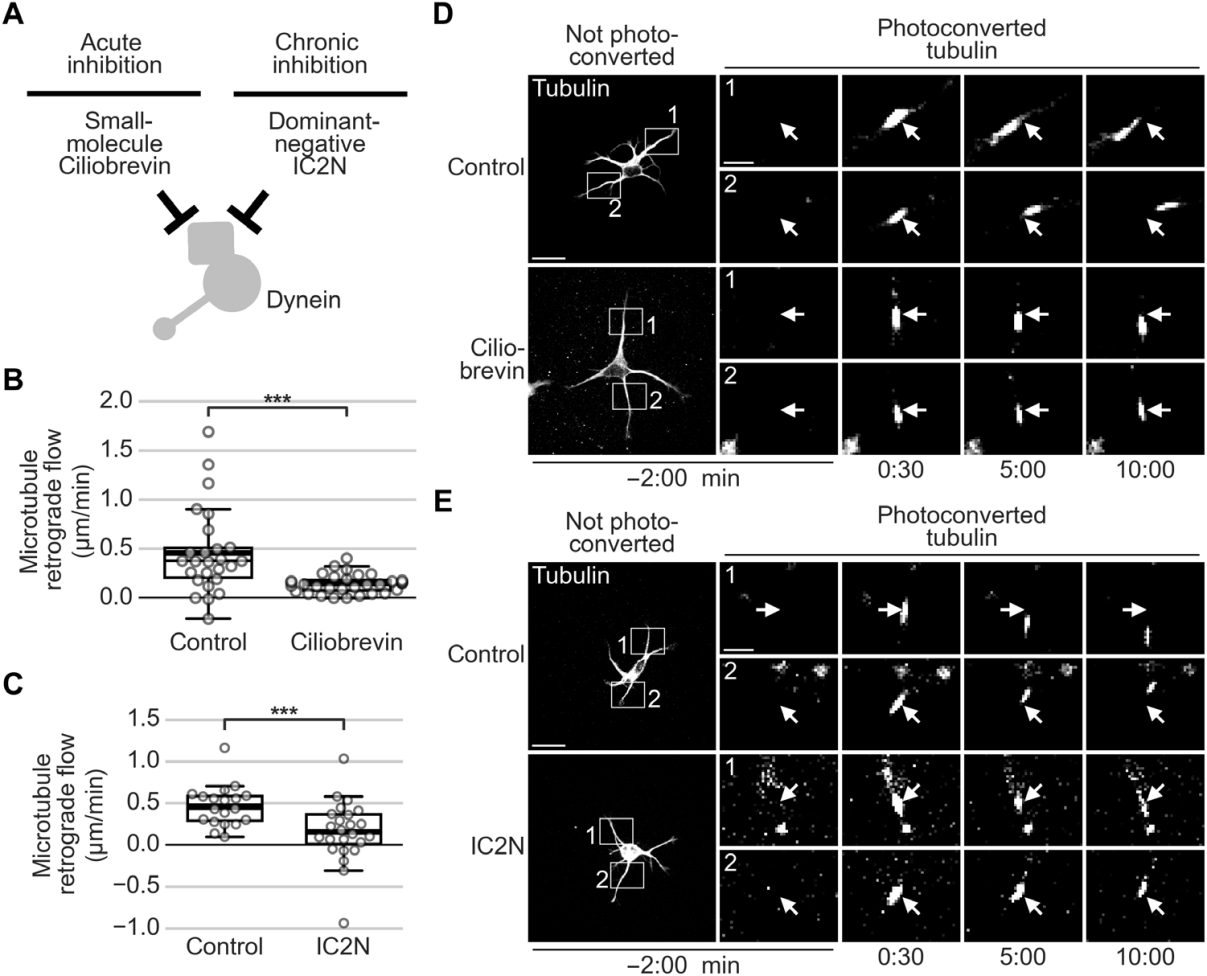
Dynein inhibition slows down MT-RF. Neurons expressed the tubulin subtype TUBB2a fused to the photoconvertible fluorophore mEos3.2 and were imaged after 1 day in culture. (**A**) Illustration of experiments. (**B**) Neurons were treated with 50 μM ciliobrevin A for 30 to 60 min and then imaged (*n* = 26 cells, *N* = 3 for control; *n* = 32 cells, *N* = 4 for ciliobrevin). (**C**) Neurons expressed the Cerulean3-labeled N-terminal 234 amino acids of the dynein intermediate chain 2 (IC2N), which inhibits dynein through the dynein-dynactin interaction (*n* = 19 cells, *N* = 3 for control; *n* = 24 cells, *N* = 4 for IC2N). (**D** and **E**) Representative neurons for (D) ciliobrevin treatment in (B) and for (E) IC2N expression in (C). Arrows indicate the location of photoconversion at 0:00 min. ***P < 0.001, Dunn’s test. Scale bars, 20 μm (overview images) and 5 μm (zoomed images).

Last, we investigated the involvement of microtubule-based motor proteins in driving MT-RF. As neurites contain microtubules in both orientations, 30% facing with their plus end toward the soma (plus-end-in) and 70% facing with their plus ends toward the tip (plus-end-out) (*29*), both minus end– and plus end–directed motors could be involved. Inhibiting the minus end–directed motor protein dynein slowed down MT-RF, both acutely following treatment with the small-molecule ciliobrevin A (*30*) and chronically following overexpression of the dominant negative–acting N-terminal domain of the dynein intermediate chain 2 (IC2N; Fig. 8, A to E, and movie S13) (*31*).

**Fig. 9.**
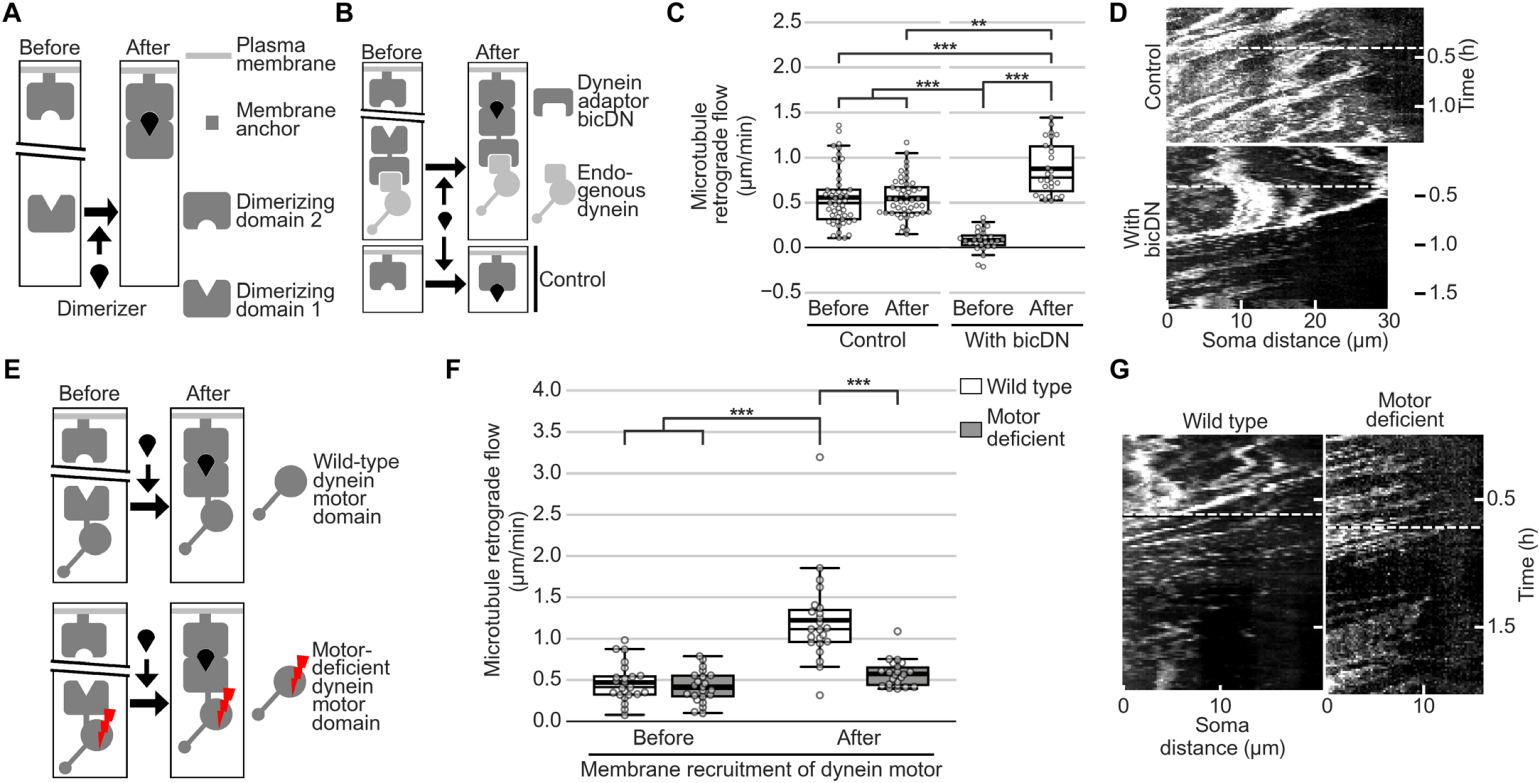
Dynein at the plasma membrane speeds up MT-RF. Neurons expressed the fluorophore mNeonGreen fused to CAMSAP3 and were imaged after 1 day in culture. (**A**) Illustration of the chemical dimerization used to recruit proteins to the plasma membrane. Dimerization domains 1 and 2 are FK506-binding protein (FKBP) and FKBP-rapamycin binding (FRB), respectively. (**B**) The N-terminal domain of the dynein adaptor bicDN (amino acids 1 to 594) was recruited to the membrane anchor CAAX in (C) and (D). (**C** and **D**) Neurons expressed FRB-Cerulean3-CAAX for control, and with bicDN additionally tdTomato-bicDN-FKBP. Neurons were imaged for 20 to 40 min, after which 0.5 μM rapalog A/C Dimerizer was added, and imaging continued. (C) Average MT-RF before and 6 min to 1 hour after dimerization was calculated from CAMSAP3 traces (for bicDN, *n* = 28 cells, *N* = 5; for control, *n* = 49, *N* = 3). (D) CAMSAP3 intensity along neurites (*x* axis) over time (*y* axis; kymograph). Dashed lines show the time at which the dimerizer was added. (**E**) Wild-type or adenosine triphosphatase (ATPase)–deficient mutated (K2599T) dynein1 motor domain (Dync1h1motor; amino acids 1453 to 4644) was recruited to the membrane anchor C2 for (F) and (G). (**F** and **G**) Neurons expressed FRB-C2-Cerulean3 with FKBP-mScarlet-Dync1h1motor (wild type) or FKBP-mScarlet-Dync1h1motorK2599T (motor deficient). Neurons were imaged for 1 to 2 hours, and then 0.5 μM rapalog A/C Dimerizer was added before continuing imaging. (F) Average MT-RF was calculated before and 6 min to 1 hour after dimerization from CAMSAP traces (*n* = 23 cells for wild-type dynein motor, *n* = 22 cells for motor-deficient dynein motor, *N* = 5 independent experiments). (G) CAMSAP3 intensity along neurites (*x* axis) over time (*y* axis; kymograph). Dashed lines show the time at which the dimerizer was added. **P < 0.01 and ***P < 0.001, Kruskal-Wallis multiple comparison with Dunn’s post hoc test with Bonferroni correction.

Dynein has diverse functions, including transporting cargo on microtubules (*32*); its global disruption induces neurite retraction through myosin II activation (*33*). Hence, global manipulation of dynein is not suitable to test the influence of MT-RF on neurons. We hypothesized that membrane-bound dynein might move microtubules and could thereby directly drive MT-RF. To test this, we used a well-established pharmacogenetic system (*34*) to recruit dynein to the plasma membrane upon addition of a dimerizer (Fig. 9A) and measured MT-RF. We started by recruiting endogenous dynein to the plasma membrane. We did this by overexpressing the N-terminal domain of the dynein adaptor bicaudal-D2 (bicDN), which binds dynein but cannot bind cargo (*35*), and then recruited bicDN itself to the plasma membrane (Fig. 9B). As bicDN inhibits dynein (*36*), expression of bicDN slowed down MT-RF. However, upon induced recruitment to the plasma membrane, MT-RF markedly increased (Fig. 9, C and D, and movie S14). We then expressed and recruited the dynein motor domain to the plasma membrane. When photoconverting microtubule patches, this recruitment moved 84 ± 5% of the photoconverted microtubule mass retrogradely, compared to 10 ± 4% anterogradely and 6 ± 2% remaining stationary (mean ± SEM; fig. S11, A and B). However, 20 to 30 min after dynein motor membrane recruitment, this speedup became less uniformly retrograde (fig. S11, C and D). Hence, dynein activity at the plasma membrane is sufficient to transiently fuel uniform MT-RF.

As a minus end–directed motor, dynein at the membrane could exert a retrogradely directed force on plus-end-in microtubules. Consistently, in the axon of neurons cultured for 7 days, which contains less than 5% plus-end-in microtubules (*29*), dynein motor membrane recruitment did not increase MT-RF (fig. S12, A to D, and movie S15). Conversely, in dendrites, which contain 50% plus-end-in microtubules (*29*), dynein motor recruitment sped up MT-RF and reduced microtubule density (fig. S12, A to D, and movie S15). This suggests that the speedup of MT-RF by dynein at the membrane depends on the presence of plus-end-in microtubules.

The speedup of MT-RF by membrane-recruited dynein was linked to the motor function of dynein. Expressing and recruiting the wild-type dynein motor domain to the membrane sped up MT-RF, while MT-RF remained unchanged upon expression and recruitment of an inactive dynein motor domain (Fig. 9, E to G, and movie S16) (*37*). We also observed MT-RF acceleration with another minus end–directed motor, KIFC1, upon recruitment to the plasma membrane (fig. S13, A to C, and movie S17). The action of KIFC1 on MT-RF also depended on a functional motor domain, as a motor-deficient mutant (*38*) did not show such a speedup.

**Fig. 10.**
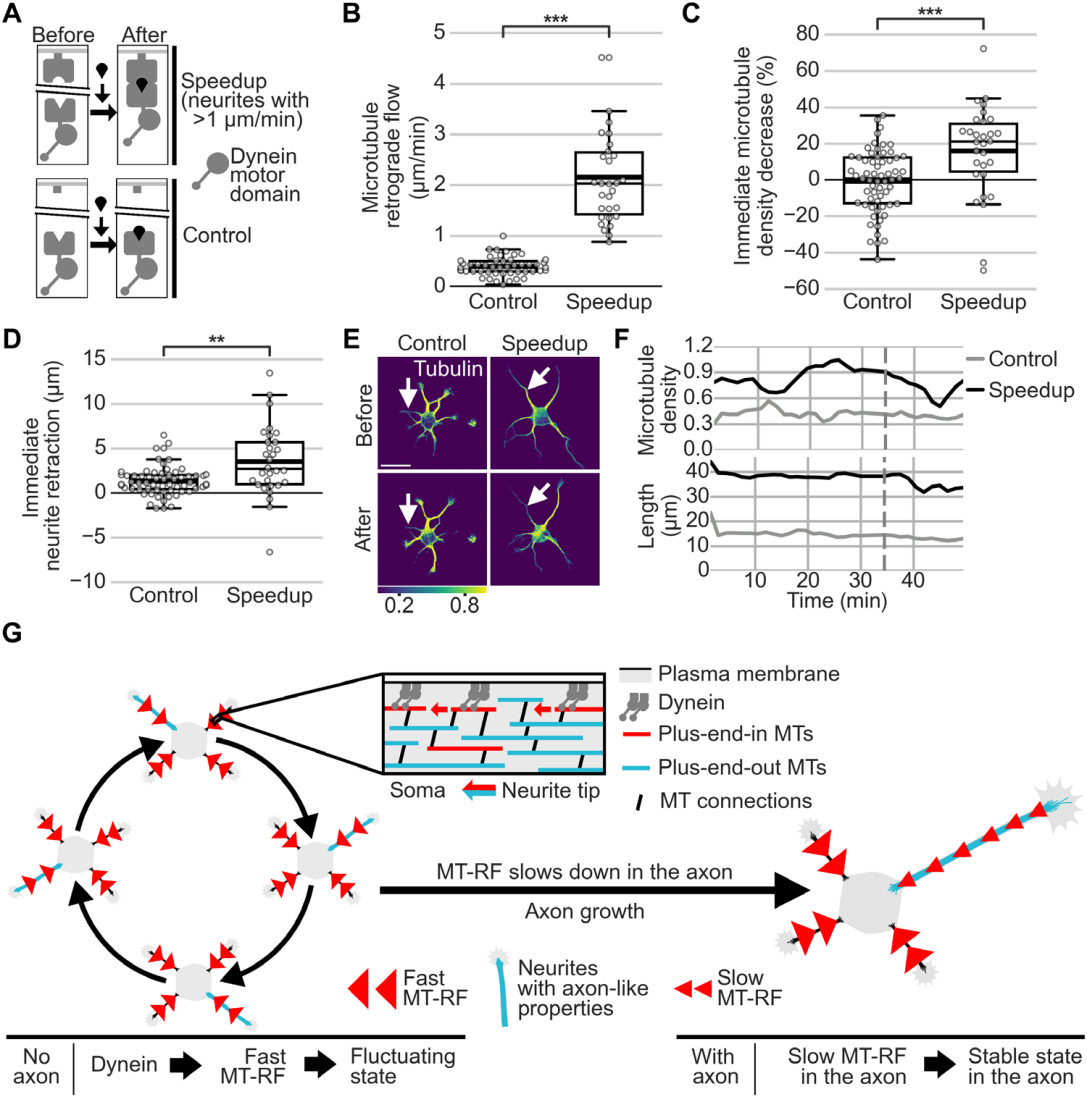
Dynein at the plasma membrane induces immediate MT density decrease and retraction. Neurons expressed the fluorophore mNeonGreen fused to CAMSAP3 and were imaged after 1 day in culture. (**A**) The wild-type motor domain of dynein1 was recruited to the membrane anchor C2 for (B) to (F). (**B** to **F**) Neurons expressed FRB-C2-Cerulean3, FKBP-Halo-Dync1h1motor, and TUBB2a-mScarlet. Neurons were imaged for 20 to 40 min, and then 62.5 nM rapalog A/C Dimerizer was added before continuing imaging. (B) MT-RF was measured from CAMSAP3 traces by averaging MT-RF from 1.5 to 15 min after dimerization. To obtain neurites with speedup from neurons with membrane-recruited dynein motor, neurites with MT-RF faster than 1 μm/min were used, while control neurites were analyzed independent of their MT-RF. Speedup was observed for membrane-recruited dynein in 86 of 368 neurites, but in 0 of 243 neurites for control. For control in (C) to (F), *n* = 62 cells; of those for (B), *n* = 48 cells, *N* = 6; for speedup, *n* = 30 cells, *N* = 5. (C) Immediate MT density decrease was calculated as the difference in normalized microtubule intensity in one neurite from before dimerization to the lowest intensity 6 to 12 min after dimerization. (D) Retraction was calculated as the difference in length of one neurite before dimerization to the smallest length 6 to 18 min after dimerization. (E) Morphology of representative cells with the neurites shown in (F) indicated by arrows. (F) Normalized tubulin intensity and length of representative neurites. (**G**) Illustration of MT-RF fueling fluctuations and then MT-RF slowing down in the axon. The thick line in boxplots shows mean. **P < 0.01 and ***P < 0.001, Dunn’s test. Scale bar, 20 μm.

Importantly, neurites with a speedup of MT-RF after recruiting the dynein motor domain to the plasma membrane (Fig. 10, A and B) showed enhanced immediate loss of microtubule density, similar to the effect found in dendrites (fig. S12C), and neurite retraction (Fig. 10, C to F, and movie S18). Thus, MT-RF keeps minor neurites short by reducing microtubule density and can thereby fuel microtubule density cycles (Fig. 10G).

The actin cytoskeleton plays a role in the speedup through the plasma membrane–recruited dynein motor domain. After actin depolymerization, recruited dynein motors moved microtubules more anterogradely, albeit a large fraction still moved retrogradely (fig. S14, A to D, and movies S19 and S20). The increase in anterograde movement was marked by a drastic increase of tubulin concentration in the distal neurite (fig. S14E and movie S20). Concomitantly, neurites rapidly extended for the next hour (fig. S14, F to H, and movie S20). This effect appears not to be caused by a change in microtubule orientation. Tracing the microtubule polymerization direction through fluorescently labeled microtubule plus-end binding 3 (EB3) protein revealed that actin depolymerization only changed microtubule orientation by 2% (fig. S15, A and B, and movie S21). In addition, pa-Blebb and taxol, both of which slowed down MT-RF, did not change microtubule orientation (fig. S15, A and B, and movie S21).

Similar to dynein and KIFC1, expression and recruitment of the plus end–directed motor protein domains of KIF1a and KIF13a to the plasma membrane also sped up MT-RF. However, upon recruitment, both kinesin motor domain constructs first moved into the soma and then briefly sped up MT-RF while also retracting neurites (fig. S16, A to I, and movie S22).

In summary, our data reveal that the microtubule array of neurites moves retrogradely toward the soma in young developing neurons. MT-RF fuels microtubule density fluctuations in neurites by reducing repeated increases in microtubule density. MT-RF subsequently slows down in the axon and thereby stabilizes microtubule density fluctuations, allowing stable axon extension. Our experiments pinpoint motor proteins as potential molecular regulators of MT-RF (Fig. 10G).

## DISCUSSION

Our work uncovers a previously unknown type of cytoskeletal dynamics, MT-RF. Through this mechanism, the microtubule array in neurites is not stationary, but instead constantly moves toward the soma during early development. Tubulin diffuses (*20*) or is transported (*21*) from the cell body to integrate into polymerizing microtubules in neurites. The whole microtubule array then retrogradely moves toward the cell body. While its mechanisms remain to be investigated, a working model of MT-RF emerges, which we describe in the following sections. We will pinpoint the questions that arise from this discovery and the implications this has for our understanding of neuronal polarization.

### Working model of MT-RF

As MT-RF is enhanced by minus end–directed membrane-bound dynein and KIFC1 motors, plus-end-in microtubules seem to play a prominent role in this process. But how can a mixed microtubule array present in neurites (*29*) enable uniform retrograde flow of the microtubule array? First, bundled and cross-linked microtubules in neurites (*2*, *39*) could transmit the force exerted on a subset of microtubules to the entire microtubule array. The bidirectional microtubule flow we observed after actin depolymerization suggests that actin can keep microtubules together for uniform retrograde flow, potentially through actin-microtubule cross-linking proteins (*7*). Such a mechanism remains to be investigated in future studies.

Then, for a net retrogradely directed force, one possibility could be that plus-end-in microtubules are not equally distributed throughout the neurite diameter, but rather concentrate along sites close to the plasma membrane. This could result in most plus-end-in microtubules close to the plasma membrane. Cryo-preserved electron microscopy and novel super-resolution fluorescence microscopy techniques (*40*, *41*) will help to characterize microtubule distribution and geometry within neurites.

Alternatively, posttranslational modifications more common on plus-end-in microtubules might make them a better substrate for minus end–directed motors. However, this preference would need to be similar for at least the two minus-end motor domains tested in this study, dynein and KIFC1, which may render this possibility less likely. Future investigations need to pinpoint the physiological location and action of the motor proteins involved in MT-RF.

We showed that pa-Blebb and taxol cause MT-RF slowdown, yet neither treatment affected microtubule orientation during this time frame, suggesting a different mechanism for this process. Taxol lowers the critical concentration for microtubule assembly (*42*) and increases the rescue rates of microtubule instability (*43*, *44*). By stabilizing microtubules, taxol also changes posttranslational modifications (*45*). Pa-Blebb is a myosin II inhibitor and decreases actin contractility (*46*–*48*) in axons and in actin arcs in the growth cone (*23*, *49*, *50*), thereby enabling microtubules to enter neurite tips more distally. Pa-Blebb also reduces neurite retraction (*51*). Myosin II might fuel MT-RF by increasing contractility in the neurite shaft or growth cone. Note that the mechanism governing how retrogradely moving microtubules are disassembled at the cell body remains to be studied. When taken together, the discovery of MT-RF opens a new field of potential research, in which there is much scope for fundamental discoveries.

### Regulation of microtubule density cycles by MT-RF

During neuronal development, microtubule density in neurites constantly cycles between low and high density, with high-density states associated with increased neurite growth (*27*). We propose that these density cycles are caused by a balance between MT-RF and microtubule polymerization. Microtubules in the neurite polymerize more than 20 times faster than the retrograde flow of microtubules (*14*, *15*). Microtubule polymerization of tubulin quickly diffusing or being transported into the neurite thereby compensates for the loss of microtubules through MT-RF. Because only some microtubules polymerize at any given time in the neurite, MT-RF and microtubule polymerization remain in a balance and keep microtubule density relatively constant. It is known that microtubule polymerization in neurites is increased in bursts during early neuronal development (*27*). When microtubule polymerization is increased, more microtubules polymerize than are lost from the neurite due to MT-RF; as a result, microtubule density increases. After this increase, microtubule polymerization decreases again and MT-RF, in turn, returns microtubule density back to baseline.

While previous models only explained the increase in microtubule density, this new model comprising MT-RF provides an explanation for the whole density cycle, including the decrease in microtubule density. This provides a basis for our understanding of neurite growth and could help pinpoint how neurites are able to explore space in search of chemical gradients for chemotaxis or connecting with neuronal and non-neuronal cells.

### MT-RF and its role during neuronal polarization

Whereas previously the tip of the neurite was thought to regulate neurite growth (*10*, *52*), our data uncover that the advance of the neurite tip is regulated by dynamics of the entire neurite, through MT-RF. We propose that MT-RF mechanically destabilizes the microtubule array to inhibit stable growth during the fluctuating state (*9*, *26*). Slowdown of MT-RF in the axon then enables the stable axonal microtubule array to be used as a scaffold for extension. Such a mechanically stable microtubule array could be required for neurite growth during neuronal polarization, as neurites of central nervous system neurons can extend without using the matrix as a scaffold on which to pull (*24*). Consequently, our work provides a paradigm shift in our understanding of neuronal polarization. While previous studies focused mainly on the axon and its growth properties (*10*, *11*), MT-RF shifts this view toward the minor neurites and that maintaining a short length is an active process.

MT-RF constitutes a previously unknown type of cytoskeletal dynamics, synchronously restructuring the cytoskeleton on an unprecedented scale by driving continuous removal of the entire microtubule mass. To maintain microtubule mass, microtubules nucleate and polymerize within the neurite (*4*, *5*). It will be interesting to see whether MT-RF also influences dynamics of microtubule-filled processes in invading carcinoma cells (*53*) and in other non-neuronal cells, including radial glia cells, developing oligodendrocytes or microglia during development, maintenance, and disease. Decades ago, the discovery of actin filaments treadmilling spawned the concept of actin retrograde flow (*54*–*56*) that radically changed the study of cytoskeleton dynamics in neurons (*52*) and non-neuronal cells (*57*). As the discovery of MT-RF is a comparable conceptual shift, we anticipate a similar boost in research.

## MATERIALS AND METHODS

### Animals

All animal experiments were performed in accordance with the Animal Welfare Act and the guidelines of the North Rhine-Westphalia State Environment Agency [Landesamt für Natur, Umwelt und Verbraucherschutz (LANUV); reference number for approval (Aktenzeichen: 84-02.04.2015.A334 and Aktenzeichen 81-02.04.2021.A208)]. Animals were group-housed (up to five mice per cage) with room temperature controlled at 21° to 22°C and an artificial 12-hour light:12-hour dark cycle (lights off at 6:00 p.m.). Mice (adult C57Bl6/J, Charles River Laboratories, JAX C57BL/6J; RRID: IMSR_JAX:000664) were given food and water ad libitum throughout the experiment.

### Neuron culture

Primary hippocampal neurons were obtained from embryonic day 16.5 (E16.5) to E17.5 mouse brains. Cells were kept at 36.5°C and 5% CO_2_. Neurons were cultured without a glia feeding layer, as previously described (*58*). After dissection, hippocampi were collected in Hanks’ balanced salt solution with magnesium and calcium (Thermo Fisher Scientific). Next, hippocampi were digested in 0.25% trypsin (Thermo Fisher Scientific, 25-200-056) at 37°C for 15 min and subsequently washed three times with minimum essential medium (MEM)–1% horse serum (MEM–1% HS) medium [1× MEM, 1× essential and nonessential amino acids, 2 mM l-glutamine (all from Thermo Fisher Scientific), 0.22% NaHCO_3_, 0.6% glucose, and 10% HS (Pan-Biotech, P30-0702)]. Last, hippocampi were mechanically dissociated with fire-polished glass-Pasteur pipettes. After transfection, 10,000 or 15,000 neurons were plated in eight-well glass-bottom dishes (ibidi, 80827-90). Dishes were coated with poly-l-lysine (1 mg/ml) at room temperature overnight, three to four times washed with double-distilled water, and placed in the incubator at 36.5°C and 5% CO_2_ with MEM–10% HS to equilibrate for at least 2 hours before plating. Two to 4 hours after plating neurons, MEM–10% HS medium was replaced by neuronal medium (N2) [1× MEM, 1 mM sodium pyruvate, 1% Neuropan 2 supplement (Pan-Biotech, P07-11010), NaHCO_3_, 0.6% glucose, and 2 mM l-glutamine], which was preconditioned through 2 to 3 days of incubation with a glia feeder layer.

### Neuron transfections

Cell transfections were performed using Nucleofector II (Lonza, AAB-1001) with program 0-005 and the mouse neuron Nucleofector Kit (Lonza, VPG-1001) according to the manufacturer’s specifications. For each transfection, 5 × 10^5^ neurons were used with varying amounts for different plasmids (table S2): 15 μg of pBetaActin-TUBB2a-mEos3.2, 15 μg of pBetaActin-TUBB2a-Dronpa, 6 μg (Fig. 3) or 3 μg (all other figures) of pBetaActin-mNeonGreen-CAMSAP3, 10 μg of pBetaActin-tandem-mCherry, 10 μg of pBetaActin-TUBB2a-mNeonGreen, 10 μg of pBetaActin-TUBB2a-mScarlet and 10 μg of pHalo-N1-TUBB5, 3 to 6 μg of pBetaActin-IC2N-dCer3, 8 μg of pBa-tdTomato-bicDN-FKBP, 10 μg of pBetaActin-FRB-dCer3-CAAX, 10 μg of pBetaActin-FRB-C2-dCer3, 10 μg of pBetaActin-dCer3-C2-dCer3, 2 μg of pBetaActin-Halo-CAMSAP3, 12 μg of pBetaActin-FKBP-mScarlet-Dync1h1motor, 12 μg of pBetaActin-FKBP-mScarlet-Dync1h1motorK2599T, 12 μg of pBetaActin-FKBP-Dync1h1motor, 12 μg of pBetaActin-FKBP-Halo-Dync1h1motor, 8 μg of pBetaActin-FKBP-mScarlet-KIFC1motor, 8 μg of pBetaActin-FKBP-mScarlet-KIFC1motorN593K, 1 μg of pBetaActin-EB3-mNeonGreen, 10 μg of pBetaActin-KIF1aMotor-mScarlet-FKBP, and 10 μg of pBetaActin-KIF13aMotor-mScarlet-FKBP. All plasmids were prepared with the EndoFree Maxiprep Kit (QIAGEN).

### Collagen 3D matrices

Neurons were grown in 3D collagen matrices as described previously at a density of 0.75 × 10^6^ to 1.5 × 10^6^ cells/ml (*24*). For the collagen matrix, 225 μl of collagen (Merck Millipore, catalog no. 08-155) with a concentration of 4.13 mg/ml was combined with 30 μl of 10× MEM and 15 μl of 5.5% sodium bicarbonate and mixed. Transfected cells in medium were added 1:3 and mixed carefully. From the hydrogel-cell mix, single 40-μl drops were added to the bottom of eight-well glass-bottom dishes (ibidi). Gels were kept in a cell culture incubator at 37°C for 20 min before the addition of 400 μl of conditioned N2 medium to fully cover gels.

### Ex utero electroporation and organotypic slice culture of embryonic brain

Ex utero electroporation and organotypic brain slices were prepared as previously described (*59*). Briefly, cortices of E14.5 embryos were electroporated with pBetaActin-TUBB2a-mEos3.2-mEos3.2-mEos3.2 (2 μg/μl) (10% FastGreen solution) by injecting DNA in both lateral ventricles using glass capillaries (prepared with a capillary electrode puller with the following settings: pressure: 500; heat: 800; pull: 30; velocity: 40) and then electroporating at an approximately 60° angle with the cathode facing the cortex (five pulses, duration of 50 ms, 30 mV, 1-s interval). Embryonic brains were then embedded in 3% low-melt melting point agarose (Biozyme, Biozym Sieve GP Agarose, 850080), and coronary slices with 180 μm thickness were prepared using a Vibratome (Leica VT1200 S vibratome). Slices were kept on polytetrafluoroethylene (PTFE) membranes (30 mm, hydrophilic PTFE, 0.4 μm; EMD Millipore, PICM0RG50) in 35-mm dishes with 1-ml slice medium (Neurobasal 1× 5% fetal calf serum, B27 supplement 1:50, GlutaMAX 1:400, penicillin-streptomycin 1:200, all from Thermo Fisher Scientific; 5% HS and Neuropan 2 supplement 1:100 from Pan-Biotech) at 35°C, 5% CO_2_ for 2 days. Directly before imaging, slices were carefully transferred into eight-well glass-bottom ibidi dishes.

### DNA constructs

pBa-tdTomato-flag-BicD2 594-FKBP (pBa-tdTomato-bicDN-FKBP) was a gift from G. Banker and M. Bentley (Addgene, plasmid no. 64205; http://n2t.net/addgene:64205; RRID: Addgene_64205) (*60*). pHalo-N1-TUBB5-Halo was a gift from Y. Okada (Addgene, plasmid no. 64691; http://n2t.net/addgene:64691; RRID: Addgene_64691) (*13*). pBetaActin-tandem-mCherry was a gift from S. Dupraz German Center for Neurodegenerative Diseases (DZNE). pBetaActin-EB3-mNeonGreen was a gift from S. Vinopal [Jan Evangelista Purkyně University in Ústí nad Labem (UJEP)].

All DNA plasmids made in this study were constructed in the pBetaActin plasmid backbone [gift from C. C. Hoogenraad (University Utrecht)] (*34*). Plasmids were constructed using homology-based cloning with the NEBuilder HiFi DNA Assembly Master Mix (New England Biolabs, #E2621L) according to the manufacturer’s specifications. Protein coding sequences were assembled by polymerase chain reaction (PCR) amplification using Q5 polymerase (New England Biolabs, #M0491S), using primers with homology sequences in overhanging ends and subsequent cleanup from an agarose gel. The plasmid backbone for these assemblies was constructed by digestion of pBetaActin plasmid with Nhe I and Hind III and subsequent cleanup from an agarose gel. One sequence was designed as a homology site for the upstream sequence in the backbone (pBetaActin upstream: CTCTGTTCCTCCGCAGCCCCCAAGCTAGC), two similar sequences for the downstream sequence (pBetaActin downstream 1: CGACTGCAGAATTCGAAGCTTCTAGA and pBetaActin downstream 2: GGTACCGTCGACTGCAGAATTCGAAGCTTTTA), and two sequences to combine protein-coding sequences together (linker 1, GGAAGTTCAGGAGGTTCTAGT, will be translated into the neutral linker sequence GSSGGSS; linker 2, TCTGGTGGATCAAGTGGTGGA, will be translated into the neutral linker sequence SGGSSGG). To combine two protein-coding sequences, only linker 1 was used, while to combine three protein-coding sequences, linker 1 was used between the first and second protein-coding sequences and linker 2 between the second and third protein-coding sequences. The same homology sequences were used for all assemblies. Primer sequences for all PCR products used for cloning plasmids can be found in table S1. The protein-coding sequence of TUBB2a was amplified from mouse cDNA. mEos3.2 was amplified from the plasmid mEos3.2-N1 (gift from M. Davidson; Addgene, plasmid no. 54525; http://n2t.net/addgene:54525; RRID: Addgene_54525) (*13*). Dronpa was amplified from the plasmid Dronpa-Tubulin-6 (gift from M. Davidson; Addgene, plasmid no. 57301; http://n2t.net/addgene:57301; RRID: Addgene_57301). CAMSAP3 was amplified from pFasBac+GFP-CAMSAP3 (gift from R. Vale; Addgene, plasmid no. 59038; http://n2t.net/addgene:59038; RRID: Addgene_59038) (*61*). MNeonGreen was amplified from the pNCS-mNeonGreen plasmid (Allele Biotechnology). MScarlet was amplified from pLifeAct_mScarlet_N1 (gift from D. Gadella; Addgene, plasmid no. 85054; http://n2t.net/addgene:85054; RRID: Addgene_85054) (*13*). pHalo-N1-TUBB5-Halo was a gift from Y. Okada (Addgene, plasmid no. 64691; http://n2t.net/addgene:64691; RRID: Addgene_64691) (*13*). C2 was amplified from CRY2clust-eGFP-2xC2-iLID (gift from D. Gadella; Addgene, plasmid no. 114420; http://n2t.net/addgene:114420; RRID: Addgene_114420) (*62*). IC2N was amplified from EGFP-IC2-N237 (gift from T. Schroer; Addgene, plasmid no. 51410; http://n2t.net/addgene:51410; RRID: Addgene_51410) (*31*). Cerulean3 was amplified from pTriEx-DORA (gift from Y. Wu) (*63*). CAAX was amplified from pLL7.0: Venus-iLID-CAAX (from KRas4B) (gift from B. Kuhlman; Addgene, plasmid no. 60411; http://n2t.net/addgene:60411; RRID: Addgene_60411) (*64*). FRB was amplified from pBa-FRB-3myc-KIF1A tail 391-1698 (gift from G. Banker and M. Bentley; Addgene, plasmid no. 64286; http://n2t.net/addgene:64286; RRID: Addgene_64286) (*60*). FK506-binding protein (FKBP) was amplified from pBa-tdTomato-flag-BicD2 594-FKBP (gift from G. Banker and M. Bentley; Addgene, plasmid no. 64205; http://n2t.net/addgene:64205; RRID: Addgene_64205) (*60*). Dync1h1 motor domain (heavy chain 1 amino acids 1453 to 4644, Dync1h1motor) was amplified from mouse cerebrum cDNA. To generate the mutated motor-deficient dynein motor domain, the correct mutation site was identified by homology analysis of protein-coding sequence for *Dictyostelium* Dynein1 and mouse Dync1h1. Mutation in *Dictyostelium* dynein1 K2675T corresponded to mouse Dync1h1 K2599. To perform the mutation, primers containing AAG to ACG mutation were designed to amplify Dync1h1motor as downstream and upstream parts, each extending 10 to 20 nucleotides over the mutation to have 20 nucleotides overlap between the two amplicons. The corresponding PCR products were used in the usual assembly strategy to directly clone pBetaActin-FKBP-mScarlet-Dync1h1motorK2599T. The mouse KIF1A motor domain (amino acids 1 to 405) was amplified from mCh-KIF1a*-strep (gift from J. Bonifacino; Addgene, plasmid no. 120165; http://n2t.net/addgene:120165; RRID: Addgene_120165) (*36*). The human KIF13A motor domain (amino acids 1 to 639) was amplified from mCh-KIF13a*-strep (gift from J. Bonifacino; Addgene, plasmid no. 120166; http://n2t.net/addgene:120166; RRID: Addgene_120166) (*36*). The human KIFC1 motor domain (amino acids 125 to 673) was amplified from Strep-KIFC1*-mCh (gift from J. Bonifacino; Addgene, plasmid no. 120169; http://n2t.net/addgene:120169; RRID: Addgene_120169) (*36*). To generate the motor-deficient KIFC1 motor domain mutant N593K (*38*), primers containing AAG to AAC mutation were designed to amplify KIFC1 as downstream and upstream parts, each extending 10 to 20 nucleotides over the mutation, so that the two amplicons had 20 nucleotides overlap between each other. The corresponding PCR products were used in the usual assembly strategy to directly clone pBetaActin-FKBP-mScarlet-KIFC1motorN593K.

### Live-cell imaging

All imaging experiments were performed on an Andor spinning disk Nikon Eclipse Ti microscope with Perfect Focus system (Nikon) and a Yokogawa CSU-X1 spinning disk unit (CSUX1-A1N-E/FB2 5000 rpm Control, FW, DMB 95L100016) using a Plan Fluor VC 40× oil objective numerical aperture (NA) 1.3 (Fig. 5, B to E, and fig. S12), Plan Apo VC 60× water objective NA 1.2 (Nikon; Fig. 2, E and F, and fig. S6), or Plan Apo VC 60× oil objective NA 1.4 with an additional 1.5 magnifier (figs. S1 and S15) or no additional magnifier (Nikon; all other figures and supplementary figures) and iQ3 software (Andor). A dichroic Quad filter (for wavelengths: 395 to 410, 482 to 492, 561 to 568, and 628 to 650) and a single band-pass emission filter for the blue channel (480/40; Semrock), the green channel (525/50; Semrock BrightLine), the red channel (617/73; Semrock BrightLine), and the far-red channel (697/75; Semrock BrightLine) were used. For most photoconversion experiments (Figs. 1, B and C, 2, B and C, 4, C and D, 6, B and C, and 8, B to E, and figs. S2, S3, S5 to S8, and S12), a quad band emission filter was used (440/40, 521/21, 607/34, and 700/45; Semrock Quad Band Filter FF01-440/521/607/700) to allow faster imaging without changing filters. For the green, red, and far-red channels, a 488-nm laser (100 mW), a 561-nm laser (100 mW), and a 640-nm laser (100 mW) were used, respectively (REVOLUTION 500 series AOTF laser modulator and combiner unit, solid-state laser modules). Experiments were conducted inside a large incubator chamber around the microscope, kept at 37°C. A small chamber for eight-well ibidi glass-bottom dishes (Pecon, #000470) was supplied with humidified air mixed with CO_2_ to a concentration of 6 to 7% CO_2_. Images were taken with an Andor iXON camera (DU897) in charge-coupled device mode at 1-MHz horizontal readout rate and with a pixel size of 0.44 μm (2 × 2 binning; Figs. 1, B and C, 2, B and C, 4, C and D, 6, B and C, and 8, B to E, and figs. S2, S3, and S5 to S8), 0.22 μm (no binning; Figs. 1, E and F, 3, B to E, 7, B to D, 9, C, D, F, and G, and 10, B to F, and figs. S4, S11, S13, S14, and S16), 0.15 μm (no binning; 1.5× magnifier; figs. S1 and S15), or 0.33 μm (no binning; 40× objective; Fig. 5, B to E, and fig. S12).

Images were either taken every 60 s (Figs. 3, B to E, 5, B to H, and 9, C and D), every 3 min (Fig. 7, B to D), every 90 s (Figs. 9, F and G, and 10, B to F, and figs. S11 to S14), every 20 s (fig. S16), every second (fig. S15), or as specified in the “Photoconversion and photoactivation” section.

For all experiments, neurons were selected on the basis of representative morphology of more than two neurites. Imaging was done under the lowest possible light exposure. Long exposure times (800 to 1000 ms) and lower laser power were preferred over shorter exposure times and higher laser power.

For drug treatments in Figs. 6 and 7 and fig. S15, 40 μM pa-Blebb [stock in dimethyl sulfoxide (DMSO); Axol Bioscience, ax494682] (*65*), 6 nM taxol (stock in DMSO; Biomol, Cay10461-25), 10 μM LatB (only for fig. S15; stock in DMSO; Merck Millipore, 428020), or DMSO (Sigma-Aldrich, D5879) as a control (all to a final DMSO concentration of 0.2%) was used. For fig. S4, 0.75 μM LatA (stock in EtOH; Sigma-Aldrich, 428021) or EtOH as a control was used. For Figs. 6 and 7, drugs were added to cells after 1 day in culture for 20 to 240 min directly before the experiment. For fig. S4, LatA or EtOH was added 240 min before imaging. For fig. S14, 10 μM LatB was added 4 to 8 hours before imaging. For fig. S15, drugs were added 3.5 to 7 hours before imaging. For drug treatments in Fig. 8 (B to E), 50 μM ciliobrevin A (Sigma-Aldrich, HPI-4, H4541) or DMSO as control was added 30 to 60 min to cells before imaging started.

### Dimerization experiments

For dimerization experiments (Figs. 9, C, D, F, and G, and 10, B to F, and figs. S11 to S14 and S16), 0.5 μM (final EtOH concentration of 0.1%; Fig. 9, C, D, F, and G, and fig. S13) or 62.5 nM (0.0125% final EtOH concentration; Fig. 10, B to F, and figs. S11, S12, S14, and S16) of the rapalog A/C Dimerizer (SiChem, SC-7000) was added after imaging for 20 to 90 min, with imaging continued after dimerization for 30 to 300 min. Images were taken every 20 s for imaging neurons with recruitment of the KIF motor domain (fig. S16) and 60 or 90 s for all other dimerization experiments.

### Photoconversion and photoactivation

For photoconversion and photoactivation experiments, the fluorescence recovery after photobleaching and photoactivation (FRAPPA) illumination system was used (Andor, Revolution). The FRAPPA module focuses the laser on single pixels for a specified time (dwell time) and moves through all pixels for a specified number of repetitions. A small area for photoconversion was chosen (specific sizes for experiments described below) to keep phototoxicity minimal.

In experiments with photoconversion of microtubule patches (Figs. 1, B and C, 2, B and C, 4, C and D, 6, B and C, and 8, B to E, and figs. S2 and S8), small microtubule patches were photoconverted in two neurites. For fig. S5, two microtubule patches were photoconverted in one axon, and for fig. S6, one microtubule patch was converted in one dendrite and one in the axon. Photoconversion was performed with the 405-nm laser (400 repetitions, 20 μs dwell time, 0.37 μW power, pixel size of 0.44 μm, 2-pixel × 1-pixel or 1-pixel × 1-pixel area). After photoconversion, images were acquired in the photoconverted (red) and not-photoconverted (green) channel every 30 s for 20 min (fig. S4) or 10 min (Figs. 1, B and C, 4, B and C, 6, B and C, and 8, B to E, and figs. S2, S3, S5, S7, and S8) or every 90 s (figs. S11, S12, and S14). For most experiments in Figs. 1 (B and C) and 2 (B and C), images of all cells were taken again 1 to 3 hours after photoconversion to check whether cells survived the experiment. Cells that died were excluded from the analysis.

For experiments with photoconversions after dynein motor domain recruitment to the plasma membrane (figs. S11, S12, and S14), photoconversion was performed with the 405-nm laser (800 repetitions, 20 μs dwell time, 0.37 μW power, pixel size of 0.22 μm, 6-pixel to 12-pixel × 1-pixel area).

In experiments with photoconversion of microtubule patches after dynein motor domain recruitment to the plasma membrane in young neurons without LatB treatment (fig. S11), CAMSAP3 was measured in addition to photoconverted microtubule patches. Microtubule patches were repeatedly photoconverted with varying time intervals once the start of MT-RF speedup in the CAMSAP3 channel was visible by eye. In LatB-treated neurons with dynein recruitment (fig. S14), photoconversion was performed 20 to 60 min before dimerization and directly after dimerization. For photoconverting microtubule patches in experiments recruiting the dynein motor domain to the plasma membrane in neurons cultured for 7 days (fig. S12), photoconversions were performed 12 to 40 min before dimerization and then repeated directly after dimerization.

In experiments with photoconversion of microtubule patches in neurons grown in 3D collagen matrices (fig. S6), photoconversion was done with the 405-nm laser (0.5 μW power, 400 repetitions, 20 μs dwell time, 2-pixel × 1-pixel area, 0.44 μm pixel size). After photoconversion, images were taken every 2 min for 10 min. *Z*-stacks were acquired at a *z*-step size from 1.7 to 1.85 μm. To mitigate the effect of retraction on measured MT-RF, we excluded neurons that showed noticeable neurite retraction. To check whether neurites retracted, the entire neuron was imaged as a larger *z*-stack following the experiment.

For photoconversion of neurons in organotypic brain slices (Fig. 2, E and F), photoconversion was done with the 405-nm laser (0.5 μW power, 400 repetitions, 20 μs dwell time, a 6-pixel to 10-pixel × 1-pixel area, 0.22 μm pixel size). Images were taken at an interval of 3 min for 30 min after photoconversion. *Z*-stacks were acquired at a *z*-step size of around 1 μm.

To test whether photoconversion reaches the entire microtubule stack (fig. S1), all neurites were photoconverted with the 405-nm laser (0.37 μW power, 400 repetitions, 20 μs dwell time, 2-pixel × 1-pixel area, 0.44 μm pixel size). *Z*-stacks were acquired with a *z*-step size of 0.25 to 0.26 μm.

In experiments where microtubule patches were photoactivated (Fig. 1, E and F, and fig. S4), two or three microtubule patches were photoactivated in a single neurite. Before photoactivation, the entire cell was bleached by taking a brief 200-ms image with high power (50%) with the 488-nm laser. Photoactivation was done with the 405-nm laser with less than 1% of the total light intensity required for photoconversion of mEos3.2 (0.04 μW power, 40 repetitions, 20 μs dwell time, 4-pixel × 1-pixel area, 0.22 μm pixel size). After photoactivation, one image was taken every 1 min for 6 min. During imaging, Dronpa gradually recovered fluorescence. To prevent background intensity from becoming too high, before every acquired frame, a part of the soma was bleached with the 488-nm laser (3.51 μW power, 10 repetitions, 20 μs dwell time, 10-pixel × 12-pixel area, 0.22 μm pixel size).

### Analysis of photoconversion in whole microtubule stack

For fig. S1, chromatic shift was corrected between the red (TUBB2a-mEos3.2; photoconverted tubulin) and the far-red channel (TUBB5-Halo; whole tubulin). The intensity in the red channel was higher than in the far-red channel when further from the cover glass. Both channels were first background subtracted. The chromatic shift was determined by measuring which *z*-slice number had the highest intensity in each channel and averaging the difference between these *z*-slice numbers across all neurites (around 1.8 *z*-slices, which was rounded to 2 *z*-slices). The average intensity for all neurites in both channels was measured in ImageJ after manual thresholding both channels to exclude neurite background. Average intensities were normalized to the highest intensity in the respective region and channel. The normalized intensities of three slices below and above the maximum slice were averaged for each channel and region. The slices with the highest normalized intensity were excluded from analysis because they are always “1.”

### Imaging of EB3 for microtubule orientation

For imaging EB3 comets to measure microtubule orientation, neurons expressing EB3-mNeonGreen were imaged once a second for 60 s before treatment. After 3.5 to 7 hours of treatment (DMSO as control, 40 μM pa-Blebb, 6 nM taxol, or 10 μM LatB), neurons were imaged again for 60 s once a second. For taxol treatment, cells were washed three times with taxol-free medium approximately 15 min before images.

### Registering low-intensity time-lapse movies (Python script livecellreg)

Time-lapse movies—used to generate kymographs (Figs. 3, B to E, 5, B to E, 7, B to D, and 9, C, D, F, and G, and 10, B to F, and figs. S11 to S14 and S16) or used for measuring MT-RF in brain slices (Fig. 2, E and F)—were registered by correcting for translation shifts with a self-made Python script. The script was developed because standard ImageJ registering plugins tested [“StackReg” (https://github.com/fiji-BIG/StackReg/blob/master/src/main/java/StackReg_.java) and “Register Virtual Stack Slices” (https://imagej.net/plugins/register-virtual-stack-slices)] did not yield satisfying results, likely because of the low intensity of registered channels. The script can register by translation in two or three dimensions. Before starting time-lapse movie registration, the maximum intensity of the movie in each pixel was set as 6 to 7 SDs of the image intensity from all time frames above the average image intensity from all time frames. This also allowed reliable registration of signals that markedly changed localization within the cell (e.g., CAMSAP3 after dynein motor domain recruitment to the membrane). To register a time-lapse movie, the correlation of a reference image with that of the frame to be registered was calculated to one pixel shift for each dimension, for eight shifts in total. The radius of this analysis successively increased by one pixel, and the direction of the shift with the best correlation value with regard to the starting shift was determined. This process was stopped when one direction of the best correlation value was obtained five times. The shift position with the best correlation value until then was used as a starting point to refine the shift values. For each dimension (*x*, *y*, and *z*) successively, the direction in which the correlation value improved was tested. If correlation improved in one direction, the shift was increased in that direction in steps of one pixel until the correlation value did not improve anymore for a defined number of pixels. This was repeated for each dimension until, at one shift value, no improvement in any dimension was found. Best shift values in *x* and *y* were used as starting shift values for the next frame; to account for gradual shifts in time-lapse movies, images were shifted by rolling them in a shift direction (numpy.roll). Reference images were rolled to avoid problems with zero values when registering low-intensity images. The reference image was changed if the correlation value using the shift from the last time frame was at least 2 SDs different from the average correlation value obtained for time points until then. This often indicated a morphological change of the neuron, which made a new reference image advantageous as it was morphologically closer to the images afterward. To register time-lapse movies from neurons in brain slices, a new reference image was additionally defined every three frames. Registered images were saved with zero values instead of rolled area in shifted areas to make the shift clearly visible. The script is available via GitHub (https://github.com/maxschelski/livecellreg).

### Kymograph generation

Time-lapse movies from which kymographs were generated were registered with a self-made Python script (see the previous section). Kymographs were generated manually in ImageJ using the KymoResliceWide plugin (https://imagej.net/plugins/kymoreslicewide) and a multiline tool with a line width of 11 to 25 pixels (Figs. 9, C, D, F, and G, and 10, B to F, and fig. S13), 11 to 40 pixels (figs. S11, S12, S14, and S16), or 11 pixels (Figs. 3, B to E, 5, B to E, and 7, B to D) and maximum intensity extracted across that width. Because of large changes in dendrite morphology after dynein motor membrane recruitment in some neurons from fig. S12, multiple kymographs were drawn over time to always cover the entire dendrite length. These kymographs were combined afterward to get one kymograph for the entire length of the experiment. Lines were oriented so that the left of the kymographs corresponded to the direction of the soma and the right to the direction of the neurite tip.

### Automated analysis of neurite length from kymographs

For Figs. 5 (B to E), 9 (F and G), and 10 (B to F) and figs. S2, S14, and S16, neurite length was automatically analyzed from kymographs using a self-made Python script. In these kymographs, rows from top to bottom corresponded to increasing time points in the time-lapse movie, while columns from left to right corresponded to points along the neurite from soma to neurite tip. For Figs. 5 (B to E) and 9 (F and G) and fig. S2, first, kymographs were smoothed (scipy.ndimage.median_filter, size = 2). For Fig. 10 (B to F) and figs. S14 and S16, kymographs were smoothed across rows [position in neurite; scipy.ndimage.median_filter, size = (1,5)] to prevent smoothening across time points (columns). Kymographs were then thresholded using a value determined manually. In thresholded kymographs, the first and last time points were always added to the thresholded area to allow for binary holes (scikit-image.morphology.binary_fill_holes). For Figs. 5 (B to E) and 9 (F and G), the thresholded image was dilated before filling holes (scipy.ndimage.binary_dilation, size = 2). The resulting area only included the neurite. Neurite length was determined for each time point as the distance from the soma to the pixel furthest from the soma that still belongs to the neurite. The last pixel that belongs to the neurite was defined as one pixel before the first not thresholded pixel closest to the soma.

### Classification of axon formation

Neurons were classified as lacking an axon when the longest neurite was shorter than 30 μm or under 5 μm longer than the second-longest neurites, as well as shorter than 50 μm. For fig. S8, neurons were considered to be at the axon transition if the longest neurite was at least 30 μm long and at least 5 μm longer than the second-longest neurite. Neurons were classified as having an axon if the longest neurite was at least 50 μm long and 10 μm longer than the second-longest neurite.

### Analysis of MT-RF

To measure MT-RF in 2D after tubulin photoconversion (Figs. 1, B and C, 2, B and C, and 4, B and C, and figs. S5, S7, and S12) or photoactivation (figs. S3 and S4), movement of microtubule patches was traced. The midpoint of microtubule patches was visually estimated, and movement along the neurite over time was traced in ImageJ using the multiline tool. Only microtubule patches that could be traced for more than 1 min were analyzed.

To analyze MT-RF in neurons without axons together with neurite growth (fig. S2), the maximum intensity along neurites (*x* axis) was plotted against time (*y* axis; kymograph). Image data from Fig. 1 (B and C) were used. Using the ImageJ line tool, the slope of the moving photoconverted microtubule patch in the kymograph was measured by dividing the width (distance) through the height (time). Length was analyzed automatically from kymographs generated from the nonphotoconverted channel, as described in the section above (see the “Automated analysis of neurite length from kymographs” section). For Fig. 1 (B and C), one neurite was excluded from analysis (speed of 3.7 μm/min) because its speed was 70% higher than the second-highest value and more than 8 SDs away from the mean.

To analyze movement of multiple microtubule patches in the same neurite (Fig. 1, D to F), movement of two to three microtubule patches in the same neurite was traced, using the same procedure as for photoconverted microtubule patches. For all combinations (up to three patches), the difference in the traveled distance was calculated. The highest traveled difference was divided by the two lower distances to obtain the relative difference in traveled distance.

To analyze MT-RF in neurons cultured in 3D collagen matrices (fig. S6) and in neurons in brain slices (Fig. 2, E and F), the movement of microtubule patches across *x*, *y*, and *z* was traced. The midpoint of patches was located by eye and marked using the ImageJ point tool for different time points in the *z*-slice, with the highest intensity of the microtubule patch. These points were used to calculate the distance the microtubule patch moved.

For measuring MT-RF with fluorescently labeled CAMSAP3 (Figs. 3, B to E, 5, B to E, 9, C, D, F, and G, and 10, B to F, and figs. S11, S13, S14, and S16), kymographs were generated (see the “Kymograph generation” section) and CAMSAP3 was traced by using the ImageJ line tool. Speed was calculated as the width (distance) divided by the height (time) of the line, whereas the angle was used to calculate direction of CAMSAP3 signals. For all analyses, only retrogradely moving CAMSAP3 signals were considered. For each time point, MT-RF speed was calculated as the median speed of all traces. To analyze slowdown of MT-RF in the axon (Fig. 3, A to E), MT-RF was smoothed with a window size of 200 min. This allowed us to exclude moments when MT-RF in another neurite slowed down to the level of the axon. MT-RF was determined to have slowed once smoothed MT-RF was 20% slower in the axon compared to all other neurites for at least 180 min.

### Fully automated script for neurite analysis (Python package “pyNeurite”)

We developed a Python package (pyNeurite; https://github.com/maxschelski/pyneurite) that traced and analyzed neurites in time-lapse movies. The package worked in multiple stages for each time frame: first, soma of the neuron was located and then the ideal threshold for the neuron was found. After that, neurites were skeletonized and traced. The last step was an analysis of intensities within neurites. The soma was extracted by using a grey opening algorithm (scipy.ndimage.grey_opening), edge extraction (scikit-image.filters.scharr), automated thresholding (scikit-image.filters.threshold_otsu), and then filling of the soma from the resultant edges (scipy.ndimage.binary_fill_holes). To obtain neuronal threshold, the lowest threshold value was found, which extracts the soma with the same size as the edge analysis. The threshold was then incrementally increased, and the thresholded image was skeletonized (scikit-image.ndimage.skeletonize). The final threshold was found a step before five or more pixels of the neurite skeleton were lost. Any gaps in neurites in the thresholded image—potentially introduced by thresholding—were closed by connecting segments detached from the soma back to soma once more. This was done by finding the point closest to the soma for each detached segment and connecting it to the closest point connected to the soma. Connections allowed were only the ones approximately in the same direction as detached segments, preventing these segments from being connected to a wrong nearby neurite, which may be closer. The dilated thresholded soma (scikit-image.morphology.binary_dilation) was removed from the resulting image, followed by skeletonization to obtain neurite skeletons. For each neurite, branch points were identified, which separated different subbranches of a neurite. Subsequently, all branches of each neurite were constructed, with each branch starting from the soma. Terminal subbranches shorter than 1 μm and neurites with less than 40 points (9 to 15 μm) were excluded. Neurites with different starting points—but overlapping in different time frames—were excluded to prevent mistakenly traced neurites from being analyzed. In addition, different branches that overlapped 70% or more in different time frames were excluded. Tracing was most challenging when a neurite was crossed by another neurite because crossing points sometimes were interpreted as branching points. By only using branch points when intensities of emerging branches were within a similar range, this error was reduced. The script only worked well with markers that label the cytosol or neurites, and not when the neurite tip was predominantly labeled (e.g., for actin or F-actin). This package is available on GitHub (https://github.com/maxschelski/pyneurite).

### Analyzing microtubule density cycles

For Fig. 5 (B to E), tubulin intensity was obtained from kymographs (see the “Kymograph generation” section) of the tubulin channel. To obtain average intensities of tubulin in neurites, kymographs were thresholded using the manually determined maximum background value. For each time point, the average intensity in the thresholded area was calculated. Instead of using kymographs for Fig. 7 (B to D), intensities were measured by a self-made fully automated Python script (see the “Fully automated script for neurite analysis (Python package “pyNeurite”)” section). For each neurite, only the branch present in most time frames was analyzed. Tubulin intensities were used (Figs. 5, B to E, and 7, B to D) to analyze microtubule density cycles using a self-made Python script. To analyze the frequency of microtubule density cycles (Figs. 5B and 7, B to D), intensity values were normalized by the average expression levels in all neurites. The average expression level was obtained by first averaging the intensity in all neurites for each time point. This average intensity over time was fitted to a quadratic function (Fig. 5, B to D) or a linear function (Fig. 7, B to D) using the polyfit function of the numpy package (numpy.polyfit) to obtain the average expression level. Intensity traces were smoothed using an averaging rolling window with a size of 2. Cycles were determined through changes of at least 0.2. One cycle consisted of an increase of 0.2 followed by a decrease of 0.2 or vice versa. The frequency of cycles was obtained by dividing the number of these cycles by the total time. For Fig. 5B, the frequency was averaged for all minor neurites of the neuron. For Fig. 7B, neurons with an axon were excluded and the frequency of microtubule density cycles was averaged for all neurites of each neuron. All speed values for one neurite were then averaged. For Fig. 5E, neurons were only considered before a neurite reached axon-like length. To correlate MT-RF with microtubule density cycles (Fig. 5E), Pearson’s *r* was calculated using the Python package SciPy (scipy.stats.pearsonr). Linear regression lines were plotted using the function lmplot from the Python package seaborn (seaborn; https://pypi.org/project/seaborn/).

For Fig. 5 (B to E), the time of axon formation was determined by using neurite length that had been automatically measured from kymographs (see the “Automated analysis of neurite length from kymographs” section). As before, an axon was defined as being at least 50 μm long and at least 10 μm longer than the second-longest neurite.

### Analysis of microtubule orientation (Python package “neuriteMT”)

Microtubule orientation was analyzed from polymerizing microtubule ends (fig. S15), labeled by expression of fluorescently tagged end-binding protein 3 (EB3). Live-cell imaging movies of EB3 were then automatically processed using the “plusTipTracker” of “u-track 2.0” in Matlab (*66*). For comet detection, a low-Gaussian SD of 1 pixel, high-pass Gaussian SD of 4 pixels, and a minimum threshold of 3 SDs with a threshold step size of 1 SD were used. For tracking, the following parameters were used: maximum gap to close was two frames, minimum length of TrackSegments from First Step was three frames, and segments were split. Cost functions and Kalman filter functions were set to “Microtubule plus-end dynamics.” U-track detected and traced EB3 comets automatically. To analyze the comet tracks further, the Python package “neuriteMT” was developed (https://github.com/maxschelski/neuritemt). This package uses all comet tracks from all time points to construct an image of the neuron. The image of the neuron was further processed using the pyNeurite package (described above) to obtain the skeletons of all neurites and the sorted points of the skeletons. The orientation of microtubules was obtained from the orientation of comet movement, relative to the orientation of neurites. For the first and last point of each comet, the closest neurite point was determined. If the last of the closest neurite points was further away or closer to the soma than the first of the closest neurite points, the comet was plus-end-out or minus-end-out, respectively. For comets where the first and last closest neurite points were the same, the direction of the comet over time was compared to the direction of the neurite skeleton at the closest neurite point. For each comet point, the closest neurite point was determined. The neurite direction at these points was then calculated from two points before up to 2 pixels after that neurite point. Comet direction was calculated at each time point, from the current time point to two time points further. The angle difference between the neurite direction and comet direction was calculated. If the angle difference was less than 70° or more than 110°, the comet at this time point was considered plus-end-out or minus-end-out, respectively. If the same orientation was determined for at least 65% of all time points, that orientation was used for the comet. If the same orientation was determined for less than 65% of all time points, no orientation was determined for the comet.

### Analysis of spread and distribution of microtubule patches

All analysis was done with kymographs from manually traced neurites. For analyzing both the spread of photoactivated microtubule patches after LatB (fig. S4) and the distribution of photoconverted microtubule patches after recruiting the dynein motor to the plasma membrane (fig. S11), first, the background intensity in the neurite was determined. This was done by taking the lowest value from the first five columns (neurite pixels) in the first and the four last rows (time points) in the kymograph. For repeated photoconversions, the neurite background from later photoconversions always had to be at least as high as the highest neurite background in previous photoconversions. This was done because, with more photoconversions, the amount of photoconverted tubulin in the neuron also increased. For both photoactivation and for each interval of photoconversion, the neurite background was subtracted from the kymograph at the corresponding interval.

Analysis of the spread of photoactivated microtubule patches after LatA treatment (fig. S4) was done by removing nonzero pixels from the edge of the kymograph at each time point after subtracting the neurite background from the kymograph. This was done by removing the pixel at the edge (anterograde or retrograde) with the lowest value until at least 10% of the summed intensity was removed. The distance between the most anterograde and retrograde pixels of the region—including the remaining 90% of the summed intensity—was the spread. To analyze the speed of anterograde and retrograde spread, the distance of the most anterograde and the most retrograde pixel in the spread from 1.1 μm retrograde and 1.1 μm anterograde of the start point of photoactivation was measured. The start point of photoactivation was determined for the first row (time point) as the pixel with the highest intensity after smoothing with a window size of 5 pixels. To obtain the speed of the spread, the row (time point) before the first with a signal lower than 1.3-fold above the neurite background was analyzed. The rapid dissipation of the photoactivated signal after LatA treatment compared to EtOH treatment led to an earlier time point being measured for LatA compared to EtOH-treated neurons.

The distribution of photoconverted microtubule patches after recruiting the dynein motor domain to the plasma membrane (fig. S11) was done by first determining the start point of the photoconversion. Without LatB treatment, the start point was determined as the pixel with the highest intensity at the first row (time point) after smoothing the intensity with a window size of 5 pixels; with LatB, the start point was directly extracted from the photoconverted regions defined during the experiment at the microscope. From that start point, the summed microtubule density (microtubule mass) after neurite background subtraction in all pixels more than 1.1 μm retrograde of the start point (retrograde), more than 1.1 μm anterograde of the start point (anterograde), or between 1.1 μm anterograde and 1.1 μm retrograde of the start point (stationary) was measured. The fraction of microtubule mass was calculated by dividing the microtubule mass in the respective region by that of the entire neurite. Only those rows (time points) up to one before the first row, with a signal of 1.8-fold (without LatB) or 1.3-fold (with LatB) above the neurite background, were analyzed. For experiments with LatB-treated neurons, the distribution was averaged from 3 to 4.5 min after dimerization. For experiments with neurons not treated with LatB, kymograph rows (time points) before speedup and up to 15 min after MT-RF speedup were analyzed. The time point of MT-RF speedup was determined manually from kymographs of CAMSAP3 along neurites.

### Analysis of proximal and distal microtubule density

The proximal and distal microtubule density after recruiting the dynein motor domain to the plasma membrane in neurons treated with LatB (fig. S14) was analyzed using kymographs of the not-photoconverted tubulin. First, the neurite was automatically thresholded (see the “Automated analysis of neurite length from kymographs” section). Within the thresholded neurite, the microtubule densities in the first 5 μm (proximal) and within 5 to 10 μm from the neurite tip (distal) were analyzed for each time point. Proximal and distal microtubule densities were normalized to the average density in the first 22.5 min and then averaged before dimerization and 30 to 60 min after dimerization.

### Analysis of KIF motor soma fraction after membrane recruitment

Soma fraction after recruiting KIF motor domains to the plasma membrane was done using ImageJ to manually create regions around the soma and the entire neuron. Images were thresholded, and the average background-subtracted intensity was multiplied with the number of pixels in the thresholded area to obtain the total fluorescence in that area for each time point. The total fluorescence in the soma region was divided by the total fluorescence in the entire neuron to obtain the soma fraction of KIF motor domains. The soma fraction was averaged over a time range of 60 to 90 min after dimerization started.

### Analysis of MT-RF from CAMSAP3 in dimerization experiments

For recruiting endogenous dynein and dynein motor domain compared to mutated dynein motor domain to the membrane (Fig. 9, C, D, F and G), average MT-RF was calculated before the addition of the dimerizer and 6 min to 1 hour after addition from CAMSAP3 traces. Cells with high bicDN expression levels or very low expression levels of membrane anchor CAAX were excluded from the analysis. For figs. S13 and S14B, MT-RF was averaged before dimerization and during the time from 3 to 30 min (fig. S13) or 30 to 45 min (fig. S14B) after dimerization. For fig. S16 (C to F), MT-RF was averaged before dimerization and at several time intervals after dimerization in relation to the time point when the soma fraction of KIF1a reached at least 25% for the first time (fig. S16, C and D) or the soma fraction of KIF13a reached at least 50% for the first time (fig. S16, E and F). Specifically, MT-RF was averaged after dimerization for the time more than 7 min before soma fraction increase (longer before), from 20 s to 7 min before soma fraction increase (right before), from 0 to 7 min after soma fraction increase (right after), and from 8 to 14 min after soma fraction increase (longer after). To check the effect of MT-RF speedup after recruiting dynein motor domain to the membrane (Fig. 10, B to F), MT-RF was averaged from 1.5 to 15 min after dimerization using CAMSAP3 traces. In neurons where dynein motor domain was recruited to the membrane, only neurites with MT-RF faster than 1 μm/min were analyzed, while for the control, all neurites were analyzed independent of MT-RF speed.

### Analysis of MT density and neurite length for dimerization experiments

MT density and neurite length were automatically extracted from kymographs of the tubulin channel (Fig. 10, C and D, and figs. S12C, S14F, and S16G). For Fig. 10C, immediate loss of MT density was obtained from the difference in normalized MT intensity from before dimerization to 4.5 to 9 min after dimerization in each neurite. For fig. S12C, the first microtubule density from the not-photoconverted tubulin channel was normalized to the average density in the first 4 min (six time frames). For fig. S14F, the growth rate was averaged from before dimerization and for the time from 30 to 60 min after dimerization. Then, microtubule density was measured in the last time frame of the video, which was 1 to 5 hours after dimerization started. For fig. S16G, retraction was calculated by comparing the neurite length to the average neurite length of 15 min before dimerization. Retraction was averaged for the time from 60 to 90 min after dimerization. Immediate retraction (Fig. 10D) was calculated by taking the difference in length before dimerization to the shortest length of 4.5 to 15 min after dimerization in each neurite. In the analysis for each neuron, values of all analyzed neurites were averaged.

### Python scripts

All scripts were written using Python 3.7 or 3.8, with environments build with conda (Anaconda Inc., 2020; available from https://docs.anaconda.com/) and using the following packages: SciPy 1.6.2 (for FigureFlow) or 1.5.2 (https://pypi.org/project/scipy/) (*67*), sci-kit image 0.17.2 or 0.18.1 (https://pypi.org/project/scikit-image/) (*68*), numpy 1.20.3 (https://pypi.org/project/numpy/) (*69*), seaborn 0.11.1 (for FigureFlow) or 0.11.0 (https://pypi.org/project/seaborn/) (*70*), scikit-posthocs 0.6.7 (https://pypi.org/project/scikit-posthocs/) (*71*), matplotlib 3.34 or 3.4.2 (https://pypi.org/project/matplotlib/) (*72*), pandas 1.3.0 (for FigureFlow) or 1.1.3 (https://pandas.pydata.org/) (*73*), and python-pptx 0.6.19 (https://pypi.org/project/python-pptx/).

### Figure and movie preparation (FigureFlow)

We developed a Python package (FigureFlow) to prepare all figures and movies. The Python package allows for a highly standardized generation of publication-grade figures and movies using simple Python scripts. The package will be published as a separate protocol and will be made installable via pip. The package is already available via GitHub (https://github.com/maxschelski/figureflow). The features include simple data plotting (e.g., automatic data plotting, calculation, and annotation of statistical data in plots), simple display of representative cells (e.g., automatic calculation of cells closest to the mean, image extraction from ImageJ Hyperstack, definition of zoom regions, and annotation of channels or time points), and automated generation of movies with the same scripts and same flexibility as figure panel generation with movie-specific features (e.g., title pages, showing specific frames longer, and repeating movie). All panels are aligned accurately according to a user-defined grid. Illustrations were made in Adobe Illustrator and Microsoft PowerPoint, while the illustration of the 3D matrix in Fig. 2D (without neurons) is from BioRender (BioRender.com).

### Statistics

For all data where more than two groups were compared, the nonparametric Kruskal-Wallis test (scipy.stats.kruskal_wallis) was first performed. If the resultant *P* value was below 0.05, all combinations of groups were compared. All statistical comparisons of the two groups were undertaken using the nonparametric Dunn’s test (scikit-posthocs.posthoc_dunn), with Bonferroni correction. If only two groups of paired data were compared (Figs. 2, B and E, 4C, and 5B and figs. S3, S5, S6, S8, S14, B and D to F, S15, and S16, B and G), then the Wilcoxon signed-rank test for paired data (scipy.stats.wilcoxon) was performed.
