# Supplementary material for: Microtubule retrograde flow retains neuronal polarization in a fluctuating state
Source: Sci Adv. 2022 Nov 4;8(44):eabo2336. doi: 10.1126/sciadv.abo2336 (PMC9635824; doi:10.1126/sciadv.abo2336)
Supplement: Supplementary file 1 — Figs. S1 to S16 Table S2 [file sciadv.abo2336_sm.pdf]

Science Advances
AAAS

Supplementary Materials for

Microtubule retrograde flow retains neuronal polarization in a fluctuating state

Max Schelski and Frank Bradke

Corresponding author: Frank Bradke, frank.bradke@dzne.de

Sci. Adv. **8**, eabo2336 (2022)
DOI: 10.1126/sciadv.abo2336

The PDF file includes:

Figs. S1 to S16
Legend for table S1
Table S2
Legends for movies S1 to S22

Other Supplementary Material for this manuscript includes the following:

Table S1
Movies S1 to S22

Supplementary Figures

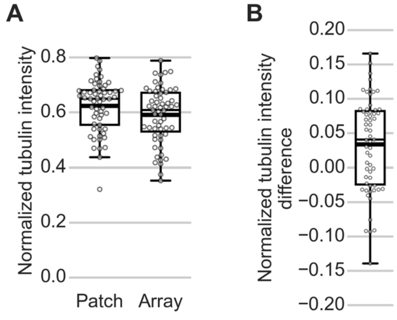

Fig. S1. Microtubules throughout the array are photoconverted.

Neurons expressing the tubulin subtype TUBB2a fused to the photoconvertible fluorophore mEos3.2, and the tubulin subtype TUBB5 fused to the halo-tag, stained with a far-red chemical fluorophore (JF646, Promega) were cultured for one day and then imaged. (**A** and **B**) After photoconversion of TUBB2a-mEos, z-stacks of the entirety of all neurites were acquired. Intensities were normalized to the slice with the highest intensity in that channel. The slices were analyzed from three z-planes further away from the glass to three z-planes closer to the glass around the highest intensity slices in both channels. The slices with the highest intensity (normalized to 1) in any channel were excluded from analysis. (**A**) The normalized intensity of each neurite was obtained as the average intensity across defined range of slices for the entire microtubule array as well as the photoconverted microtubule patch. Comparison of the normalized intensity for microtubule patch (photoconverted TUBB2a-mEos) and microtubule array (TUBB5-Halo stained with JF646) is shown. (**B**). The difference of normalized intensity was calculated for each slice separately and then averaged across all slices for each microtubule patch. (55 neurites, 14 cells, N = 2 independent experiments). Thick line in boxplots represents the mean. p value > 0.05, Dunn's test.

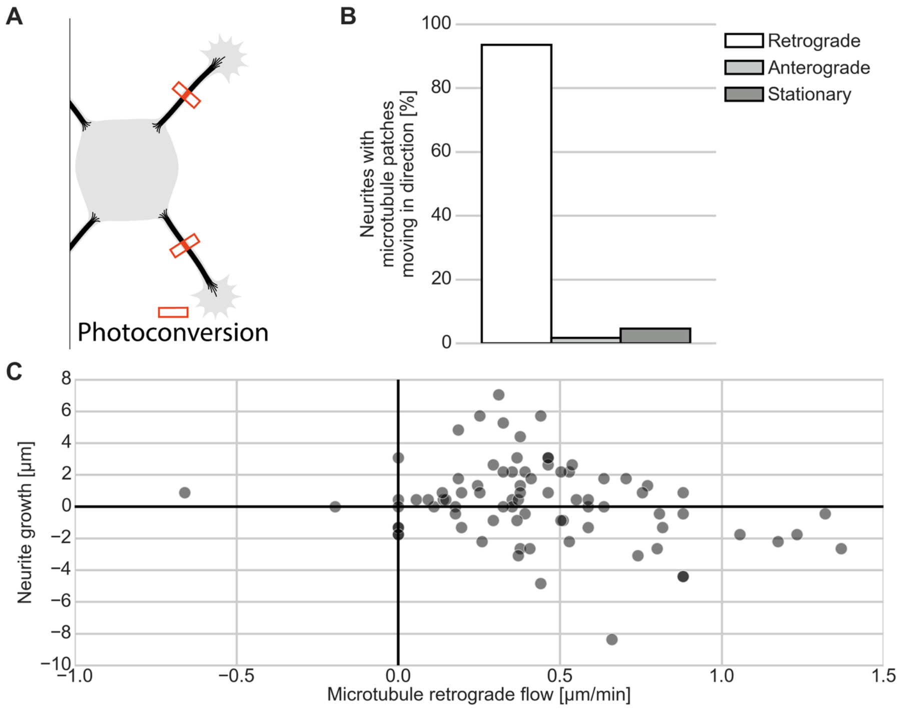

Fig. S2. Microtubules flow retrogradely in neurons in absence of neurite retraction. Neurons expressing the tubulin subtype TUBB2a fused to the photoconvertible fluorophore mEos3.2 were cultured for one day and then imaged. (A) Illustration for photoconversion experiment in [B and C]. (B) Percentage of neurites with microtubules moving retrogradely (towards the soma), anterogradely (towards the neurite tip) and not moving from total neurites imaged. Data from Fig. 1B (n = 71 cells, N = 11 independent experiments). (C) Microtubule retrograde flow (x-axis) plotted against neurite growth during 10 min imaging intervals (y-axis). Part of image data from Fig. 1B was reanalyzed to measure neurite growth and microtubule retrograde flow together. (n = 42 cells, N = 10 independent experiments).

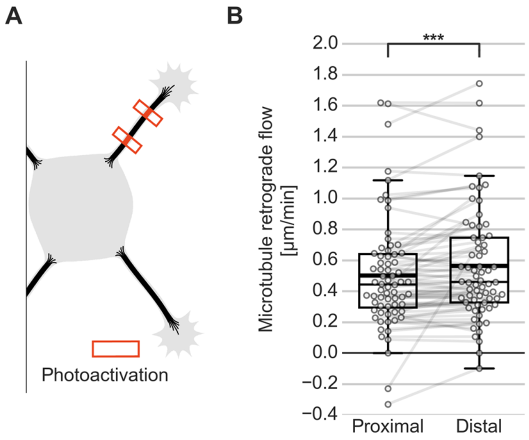

Fig. S3. MT-RF is similar in proximal and distal parts of the neurite.

Neurons expressing the tubulin subtype TUBB2a fused to the photoactivatable fluorophore Dronpa were imaged after one day in culture. (**A**) Illustration of the photoactivation experiment. (**B**) MT-RF measured for the distal and proximal photoactivated microtubule patches. Images used for analysis were the same as for [Fig. 1, E and F] (n = 68 cells, N = 7 independent experiments). Thick line in boxplots represents the mean. ***P < 0.001, Wilcoxon signed-rank test.

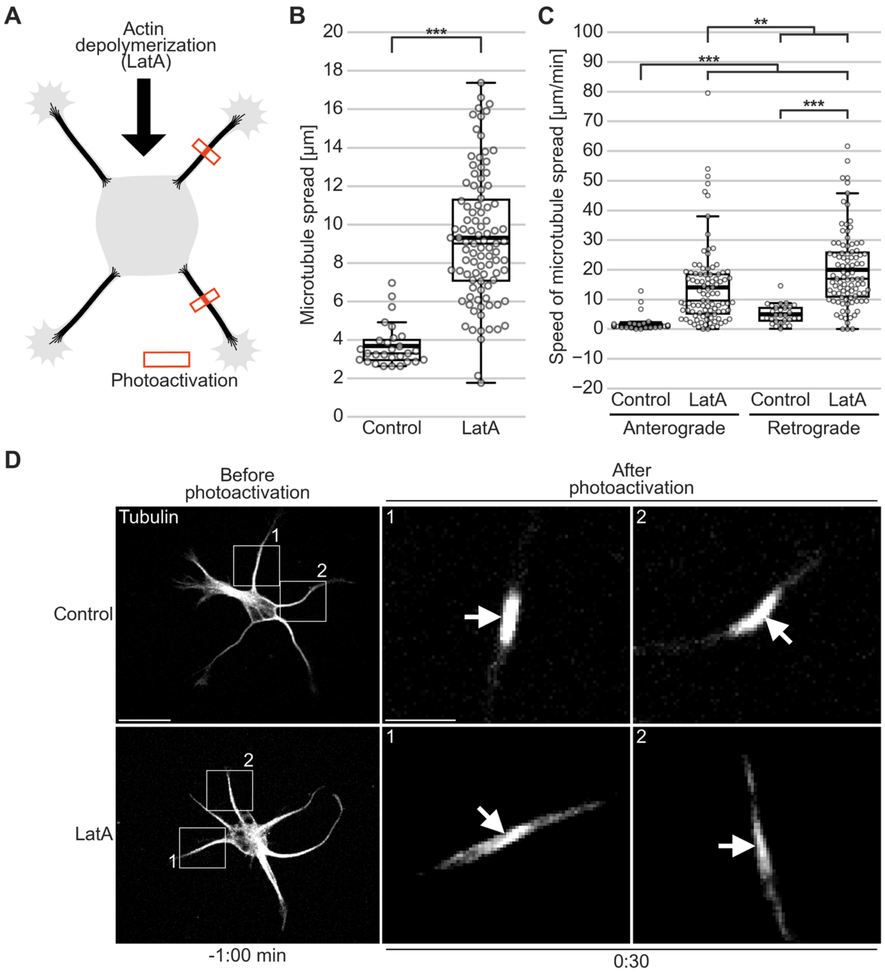

Fig. S4. After actin depolymerization microtubules move quickly in both directions. Neurons expressing the tubulin subtype TUBB2a fused to the photoactivatable fluorophore Dronpa were cultured for one day, treated with 0.75 µM latrunculin A (LatA) for four hours and then microtubule patches were photoactivated and imaged for up to 10 minutes. (**A**) Illustration of experiment. (**B** and **C**) Spread of photoactivated tubulin (microtubule spread) was measured as the length around the point of photoactivation that includes 90% of photoactivated tubulin mass. (**B**) The length of the spread. (**C**) The distance of the furthest anterograde and retrograde point of the spread from 1.1 µm anterograde and retrograde of the photoactivation point was measured. This distance was divided by the time since photoactivation to obtain the speed of anterograde and retrograde spread. (n = 28 cells for control, N=2; n=95 cells for LatA, N = 3; N, number of independent experiments). (**D**) Representative neurons with photoactivation done at

0:00 min. Scale bar, 20 μm for overview images, 5 μm for zoomed images. Thick line in boxplots represents the mean. **$P < 0.01$, ***$P < 0.001$, Wilcoxon signed-rank test for [B] and Kruskal Wallis multiple comparison with Dunn's posthoc test with Bonferroni correction for [C].

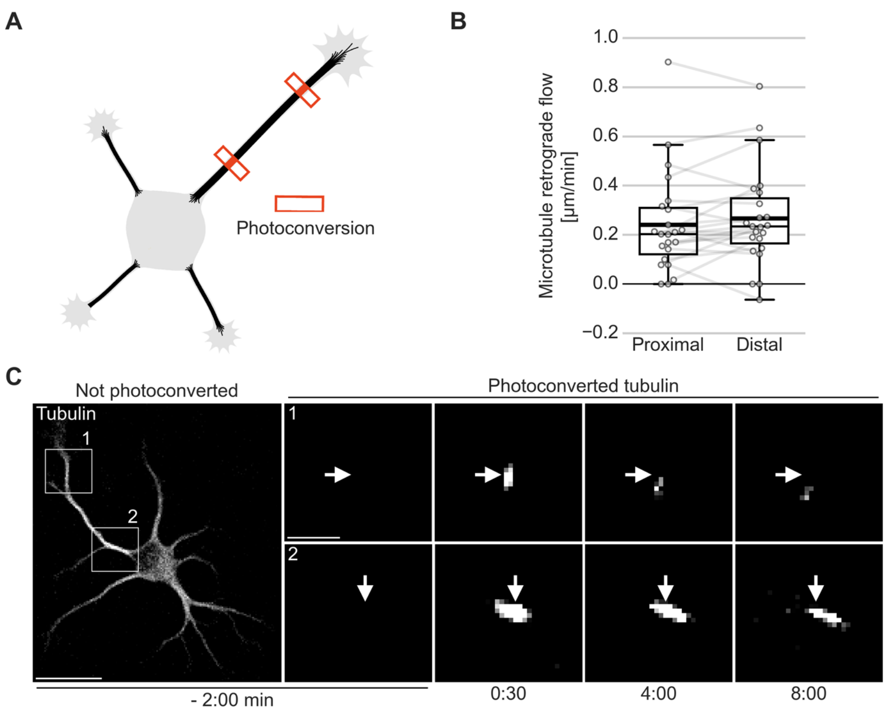

Fig. S5. MT-RF is similar in proximal and distal parts of the axon.

Neurons expressing the tubulin subtype TUBB2a fused to the photoconvertible fluorophore mEos3.2 were imaged after one day in culture. (**A**) Illustration of the photoconversion experiment. (**B**) MT-RF of the distal and proximal photoconverted microtubule patches. Only neurons with at least one microtubule patch moving were analyzed. (n = 23 cells, N = 4 independent experiments). Thick line in boxplots represents the mean. (**C**) Representative cell for [B]. Photoconversion was done at 0:00 min and white arrows point to the site of photoconversion. Scale bar, 20 μm for overview image, 5 μm for zoomed images. All p-value > 0.05, Wilcoxon signed-rank test.

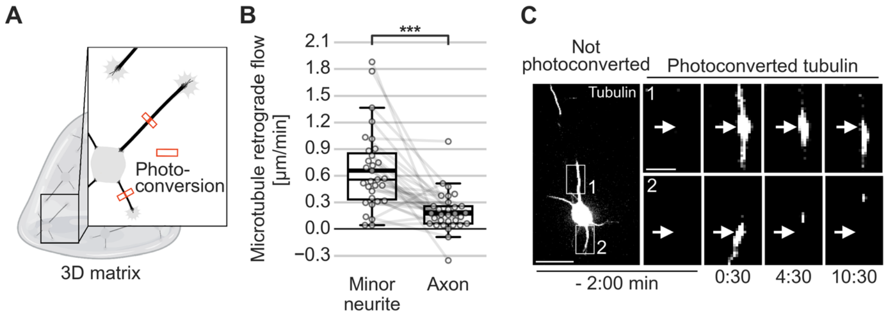

Fig. S6. In neurons grown in 3-dimensional matrices, MT-RF slows down in the axon.

The tubulin subtype TUBB2a was fused to the photoconvertible fluorophore mEos3.2, expressed in neurons and then imaged in neurons after one day in culture. (**A**) Illustration of photoconversion experiment for [B and C]. (**B**) MT-RF of neurons with axon grown in 3D. Patches that moved anterogradely were plotted with zero MT-RF. Thick line in boxplots shows mean. (**C**) Maximum intensity projection of representative cell from [B] with photoconversion done at 0:00 min and white arrows pointing to the site of photoconversion (n = 31 cells, N = 4 independent experiments). ***$P < 0.001$, Wilcoxon signed-rank test. Scale bar, 20 µm for overview image, 5 µm for zoomed images.

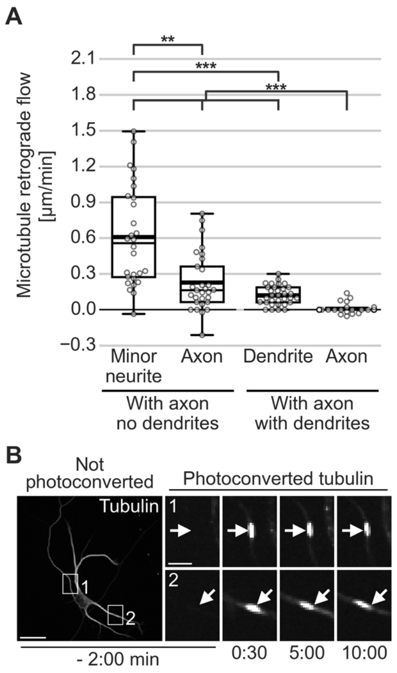

Fig. S7. Later in development MT-RF slows down in dendrites and axons.

The tubulin subtype TUBB2a was fused to the photoconvertible fluorophore mEos3.2, expressed in neurons and then imaged after one day ("With axon, no dendrites") or after 6-to-7 days ("With axon, with dendrites"). (**A**) Quantification of MT-RF. Data from the condition "With axon, no dendrites" are the same as in [Fig. 2B] (n = 31 cells for neurons without dendrites, of those n = 29 cells for axons and n = 28 cells for minor neurites, N = 8 independent experiments; and n = 32 cells for neurons with dendrites, N = 2 independent experiments). Thick line in boxplots represents the mean. (**B**) Representative neuron with dendrites for [A] with photoconversion done at 0:00 min and white arrows pointing to the site of photoconversion. **P < 0.01, ***P < 0.001, Kruskal Wallis multiple comparison with Dunn's posthoc test with Bonferroni correction. Scale bar, 20 µm for overview image, 5 µm for zoomed images.

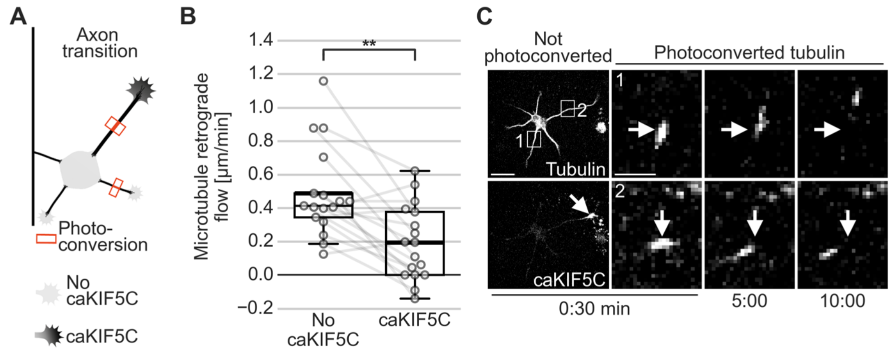

Fig. S8. MT-RF slows down in neurites with caKIF5C at the axon transition.

The tubulin subtype TUBB2a was fused to the photoconvertible fluorophore mEos3.2 and caKIF5C was fused to the fluorophore Cerulean3, expressed in neurons, and then imaged after one day. (**A**) Illustration of photoconversion experiments for [B and C]. (**B** and **C**) MT-RF in neurites without and with caKIF5C accumulation in neurons at the phase of axon growth. Neurons where the longest neurite was at least 30 µm long and at least 5 µm longer than the second -longest neurite were considered to be at the phase of axon growth ($n = 17$ cells, $N = 7$ independent experiments). White arrows in the photoconverted channel of [C] indicate areas of photoconversion and in the caKIF5C channel points to the growth cone with caKIF5C accumulation. Thick line in boxplots represents the mean. **$P < 0.01$, Wilcoxon signed-rank test. Scale bar, 20 µm for overview images, 5 µm for zoomed images.

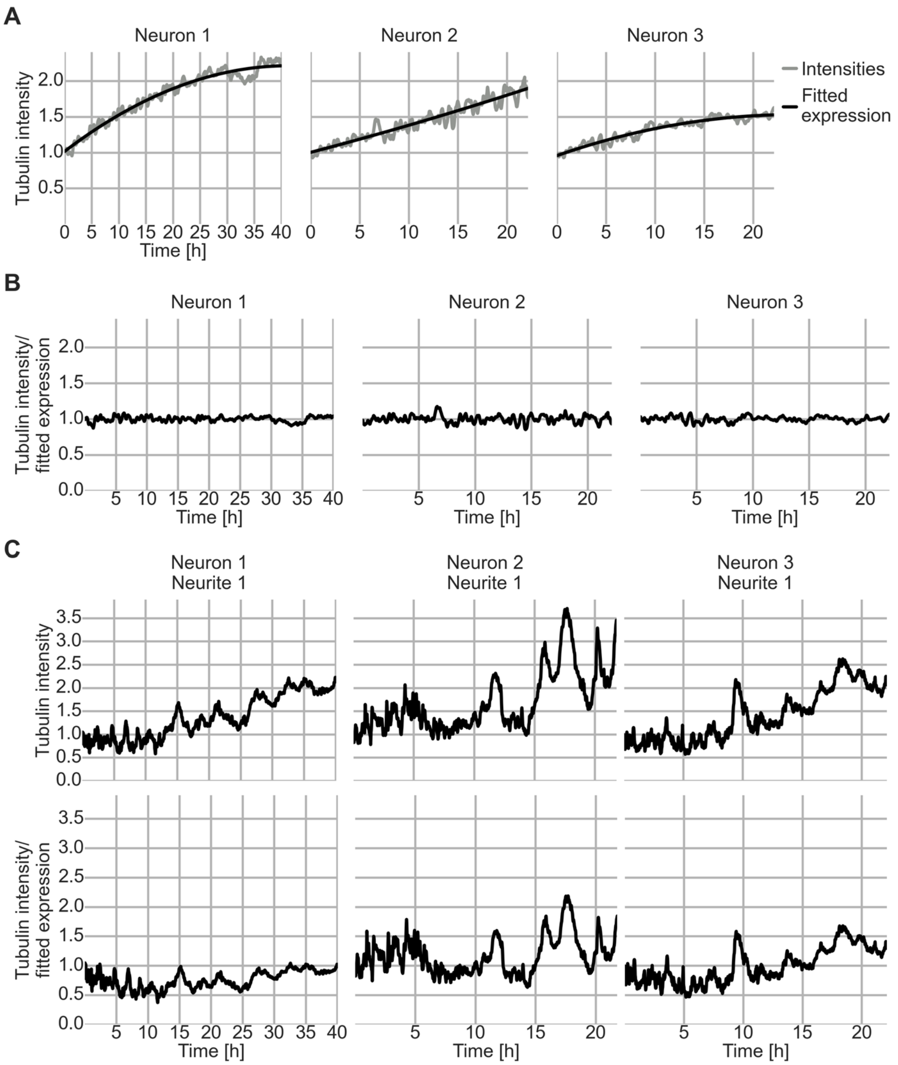

Fig. S9. Microtubule density increases due to increasing expression levels, which can be corrected for measuring density cycles.

Data shown is from dataset in [Fig. 5, B to E]. Neurons expressed the tubulin subtype TUBB2a fused to the fluorophore mScarlet and were imaged after one day in culture from before to after axon formation. (A) Average tubulin intensities in all neurites normalized to the first timeframe and the quadratic function fitted to the tubulin intensity over time normalized to the first timeframe. (B) Average tubulin intensities in all neurites divided by the fitted expression. (C) Average tubulin intensities and average tubulin intensities divided by the fitted expression for one neurite of each example neuron in [A and B].

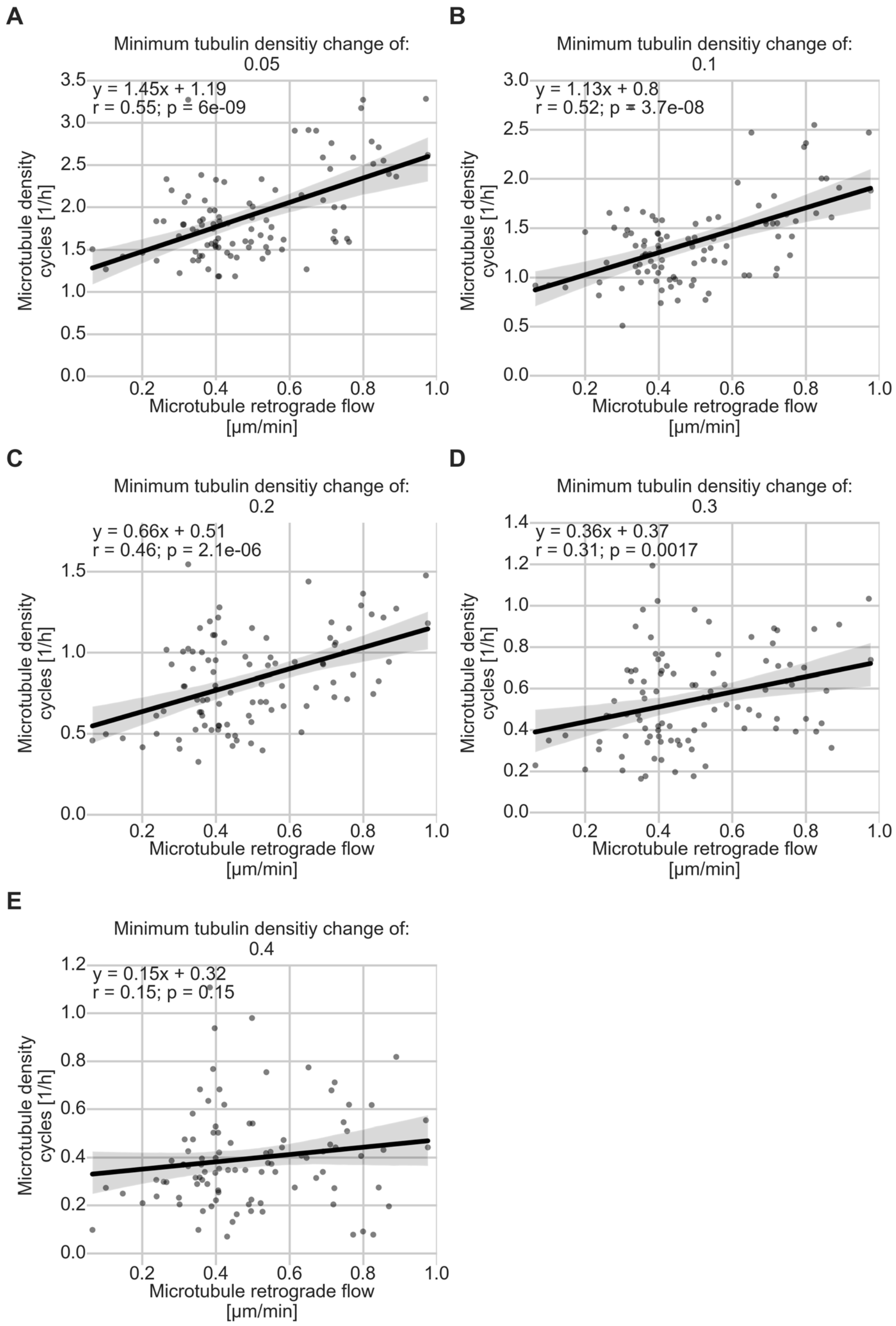

Fig. S10. The correlation of microtubule density cycles with MT-RF is robust.

Data from [Fig. 5E]. Neurons expressing the fluorophore mNeonGreen fused to CAMSAP3 and the tubulin subtype TUBB2a fused to the fluorophore mScarlet and were imaged after one day in culture. Microtubules and MT-RF were imaged simultaneously in neurons before axon formation. Microtubule density was calculated by dividing neuronal tubulin expression by average tubulin intensity, modelled as a quadratic function

of the average intensity in all neurites pooled for each timepoint. A change of microtubule density in a neurite of (**A**) 0.05, (**B**) 0.1, (**C**) 0.2, (**D**) 0.3 (**E**) 0.4 or more is half a cycle. An increase followed by a decrease, or vice versa, completes a full cycle.

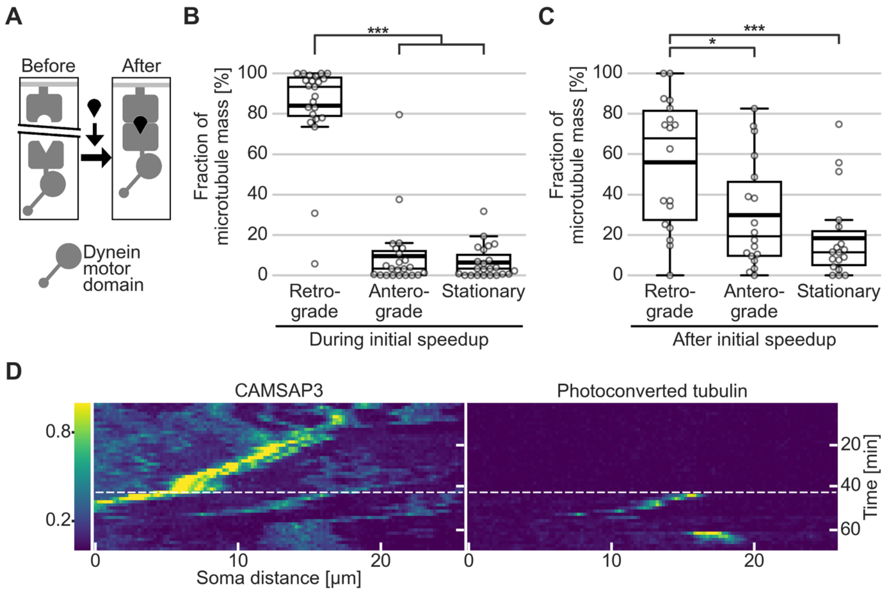

Fig. S11. Dynein motor domain recruitment to the membrane transiently moves microtubules retrogradely.

The dynein1 motor domain (dynein1 heavy chain 1 amino acids 1453 to 4644, Dync1h1motor) was recruited to the membrane anchor C2 with 62.5 nM of the rapalog A/C Dimerizer. Neurons expressed the tubulin subtype TUBB2a fused to the photoconvertible fluorophore mEos3.2, HaloTag fused to CAMSAP3, FRB-Dync1h1motor and FRB-C2-Cerulean3. HaloTag was stained with the fluorophore JaneliaFluor 646 to visualize CAMSAP3. The time of MT-RF speedup after dynein motor domain recruitment was determined manually from the CAMSAP3 signal. The distribution of photoconverted microtubules during speedup of MT-RF was measured. (**A**) Illustration of the dimerization experiment. (**B** and **C**) The distribution of photoconverted microtubule mass was measured (**B**) up to 10 min after speedup and (**C**) after 10 min. Photoconverted tubulin intensity more than 1.1 µm anterograde and more than 1.1 µm retrograde of the point with the highest intensity in the first frame was considered anterograde and retrograde, respectively. Intensity between these two points was considered stationary. (For [B] n = 23 cells, N = 6; for [C] n=18 cells, N=5; N, number of independent experiments) (**D**) Intensity of HaloTag-CAMSAP3 and photoconverted tubulin along the neurite (x-axis) over time (y-axis; kymograph). The left side is close to the soma. A trace from top right to lower left is MT-RF. The dashed line indicates the time right before MT-

RF speedup. Color-coded intensities shown (blue lower, yellow higher intensity). Thick line in boxplots represents the mean. *P < 0.05, ***P < 0.001, Kruskal Wallis multiple comparison with Dunn's posthoc test with Bonferroni correction.

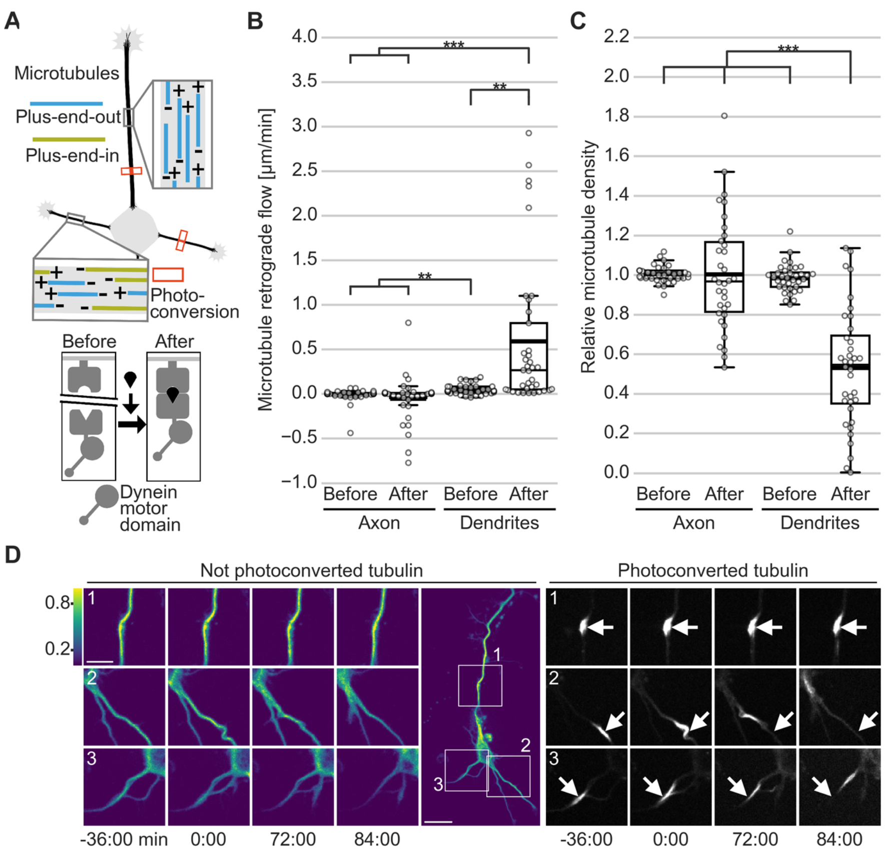

Fig. S12. Dynein motor domain recruitment to the membrane in old neurons speeds up MT-RF and leads to microtubule loss in dendrites but not in axons.

The dynein domain (dynein1 heavy chain 1 amino acids 1453 to 4644, Dync1h1motor) was recruited to the membrane anchor C2 after photoconversion of microtubule patches in neurons cultured for seven days. Neurons expressed the tubulin subtype TUBB2a fused to the photoconvertible fluorophore mEos3.2, FKBP-Halo-Dync1h1motor and FRB-C2-dCerulean3. Microtubule patches were photoconverted and neurons were imaged for 12 to 40 min, then microtubule patches were photoconverted again, 62.5 nM of the rapalog A/C dimerizer were added and neurons were imaged further for one hour to several hours. (**A**) Illustration of the dimerization experiment. (**B**) MT-RF was measured at the last timepoint before the end of the movie, before the microtubule patch entered the soma or before it dissolved. MT-RF was averaged for all dendrites of one neuron. (n = 36 cells for axon, n=37 cells for dendrite, N = 3 independent experiments) (**C**) Tubulin density from the not photoconverted channel was normalized to the average density in

the first 6 timeframes and then measured up to 5 h after dimerization started, at the last timeframe of the video (n = 34 cells for axon, n=36 cells for dendrite, N = 3 independent experiments) (**D**) Color coded tubulin density (blue lower, yellow higher intensity) and photoconverted tubulin of a representative cell with dimerization at 0:00 min. Scale bar, 20 μm for overview image, 5 μm for zoomed images. Thick line in boxplots represents the mean. $^{**}P < 0.01$, $^{***}P < 0.001$, Kruskal Wallis multiple comparison with Dunn's posthoc test with Bonferroni correction.

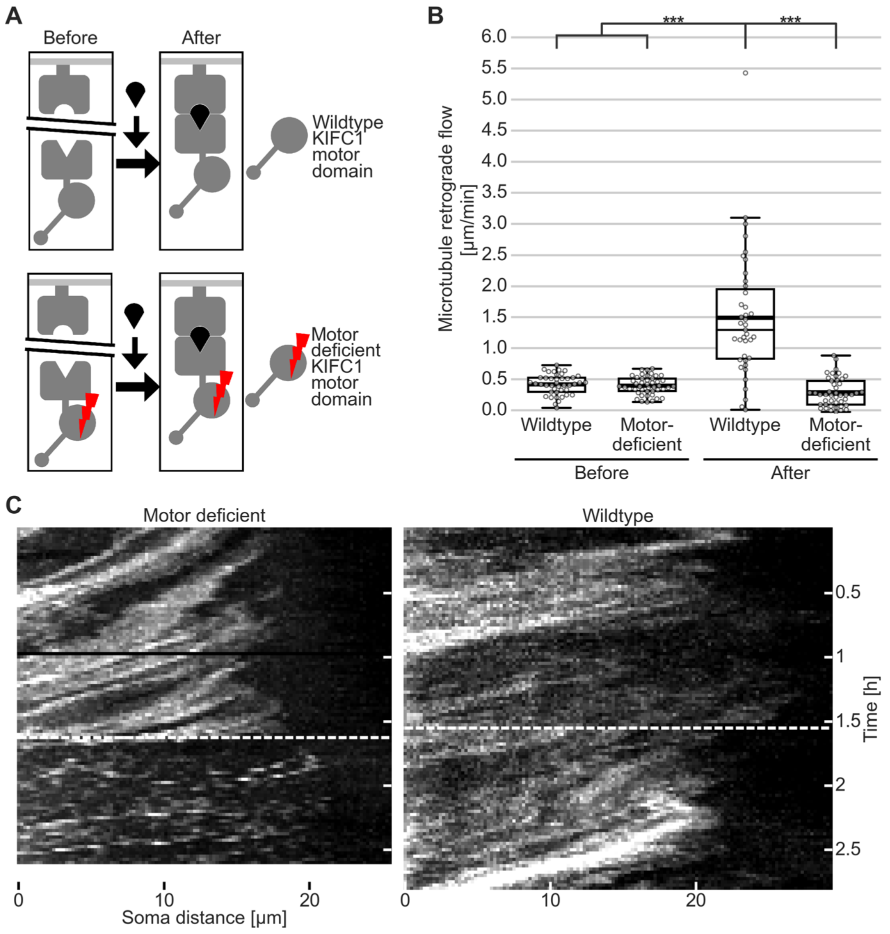

Fig. S13. Recruiting the minus-end directed motor KIFC1 to the plasma membrane speeds up MT-RF.

The wildtype motor domain of KIFC1 (amino acids 125-673) or a motor deficient KIFC1 motor domain (amino acids 125-673, N593K) was recruited to the membrane anchor C2. Neurons expressed the fluorophore mNeonGreen fused to CAMSAP3, FKBP-mScarlet-KIFC1 and FRB-C2- dCerulean3 and were imaged after one day in culture. (**A**) Illustration of the dimerization experiment. (**B**) Neurons were imaged for one to two hours, then 0.5 µM of the rapalog A/C dimerizer was added to start the recruitment. Imaging was continued for approximately one more hour. MT-RF was analyzed from 3 to 30 min after dimerization. (n = 45 cells for motor deficient KIFC1, n=42 cells for wildtype KIFC1 before and n=39 for wildtype KIFC1 after, N = 5 independent experiments) Thick line in boxplots represents the mean. (**C**) mNeonGreen-CAMSAP3 intensity along neurites (x-axis) over

time (y-axis; kymograph). The left side is closer to the soma and therefore a line from top right to bottom left shows MT-RF. The white dashes line indicates the timepoint of dimerization. ***P < 0.001, Kruskal Wallis multiple comparison with Dunn’s posthoc test with Bonferroni correction.

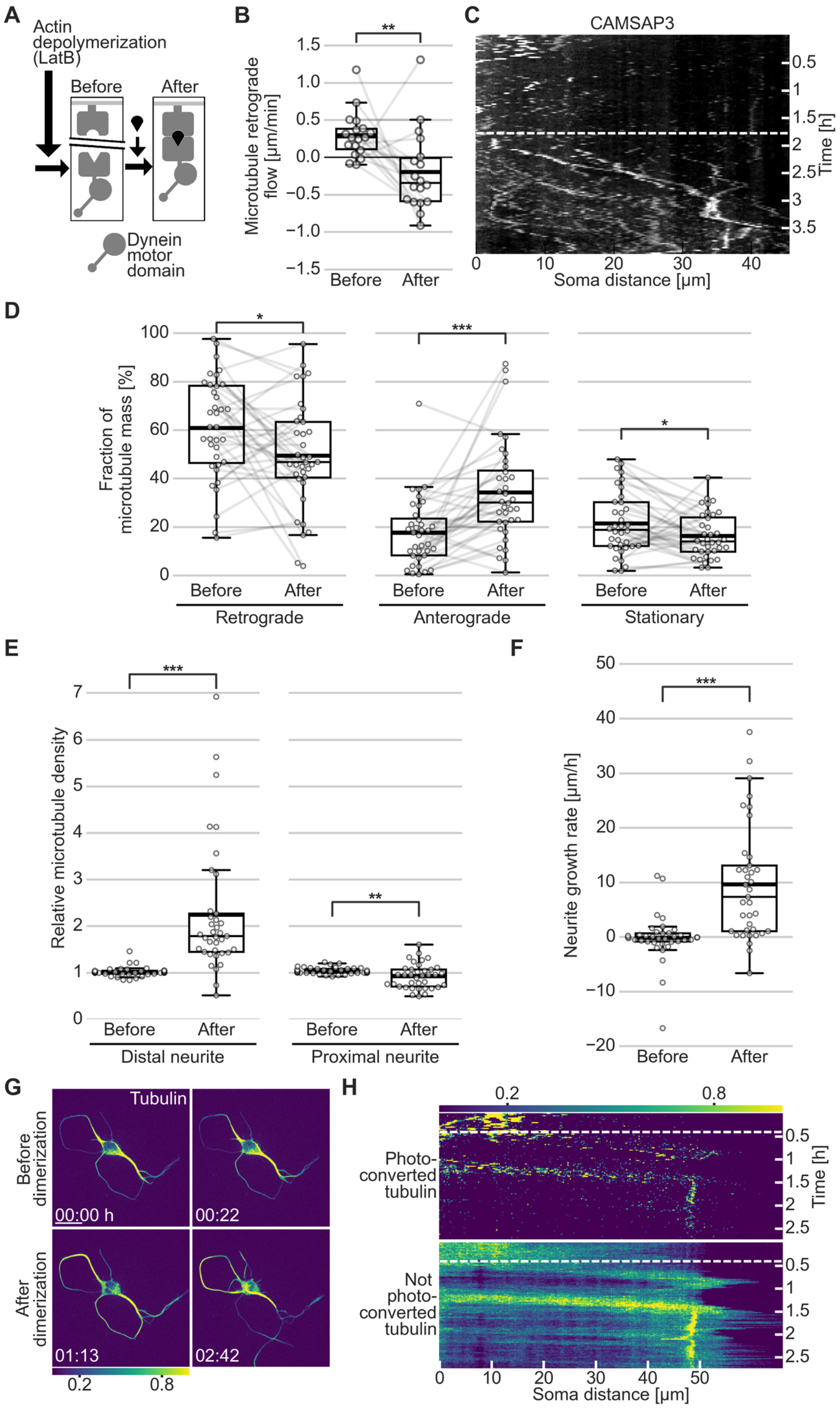
A
Actin depolymerization (LatB)
Before
After
Dynein motor domain
B
Microtubule retrograde flow [µm/min]
**
Before
After
C
CAMSAP3
Time [h]
Soma distance [µm]
D
Fraction of microtubule mass [%]
*
***
*
Before
After
Retrograde
Anterograde
Stationary
E
Relative microtubule density
***
**
Before
After
Distal neurite
Proximal neurite
F
Neurite growth rate [µm/h]
***
Before
After
G
Tubulin
Before dimerization
After dimerization
00:00 h
00:22
01:13
02:42
0.2
0.8
H
0.2
0.8
Photo-converted tubulin
Not photo-converted tubulin
Time [h]
Soma distance [µm]

Fig. S14. Recruiting the dynein motor domain to the plasma membrane after actin depolymerization leads to anterograde microtubule flow and neurite growth.

The dynein domain (dynein1 heavy chain 1 amino acids 1453 to 4644, Dync1h1motor) was recruited to the membrane anchor C2 after 4 to 8 h treatment with 10 µM latrunculin B. Neurons expressed FKBP-Halo-Dync1h1motor, FRB-C2-dCerulean3 and (B and C) the fluorophore mNeonGreen fused to CAMSAP3 or (D to H) the tubulin subtype TUBB2a fused to the photoconvertible fluorophore mEos3.2. Neurons were imaged after one day in culture (B and C) for 45 to 105 min or (D to H) 24 to 54 min, then 62.5 nM of the rapalog A/C dimerizer were added and imaging was continued. (**A**) Illustration of the dimerization experiment. (**B**) MT-RF was measured from CAMSAP3 traces before dimerization and from 30 to 60 min after dimerization. (n = 18 cells, N=2 independent experiments) (**C**) CAMSAP intensity along neurites (x-axis) over time (y-axis; kymograph). The dashed line indicates the time of dimerization. (**D** to **F**) Before and directly after dimerization, microtubule patches were photoconverted and imaged. (**D**) Photoconverted tubulin more than 1.1 µm anterograde and more than 1.1 µm retrograde of the photoconversion point was averaged from 3 to 4.5 min after photoconversion, before and after dimerization. The fraction of microtubule mass before and after dimerization were compared. (**E**) Tubulin density was measured in the proximal 5 µm and the region of 5 to 10 µm from the distal end of each neurite and normalized to the average density in the respective region in the first 22.5 min. Relative microtubule density was averaged before dimerization and from 30 to 60 min after dimerization. Relative microtubule density was compared before and after dimerization. (**F**) Neurite growth rate was averaged over all timepoints before dimerization and for 0 to 30 min after dimerization. (For [E and F] n = 37, of those n = 36 cells for [D]; N = 5 independent experiments) (**G**) Color coded tubulin density (blue lower, yellow higher intensity) of a representative cell before and after dimerization at 00:24 h. Scale bar, 20 µm. (**H**) Color coded photoconverted and not photoconverted microtubule density (blue lower, yellow higher intensity) along neurite (x-axis) over time (y-axis; kymograph) for one neurite from [G]. The soma is close to the left side. A trace from top right to bottom left indicates retrograde movement while a trace from top left to bottom right indicates anterograde movement. The dashed white line indicates the time of

dimerization. Thick line in boxplots represents the mean. *P < 0.05, **P < 0.01, ***P < 0.001, Wilcoxon signed-rank test.

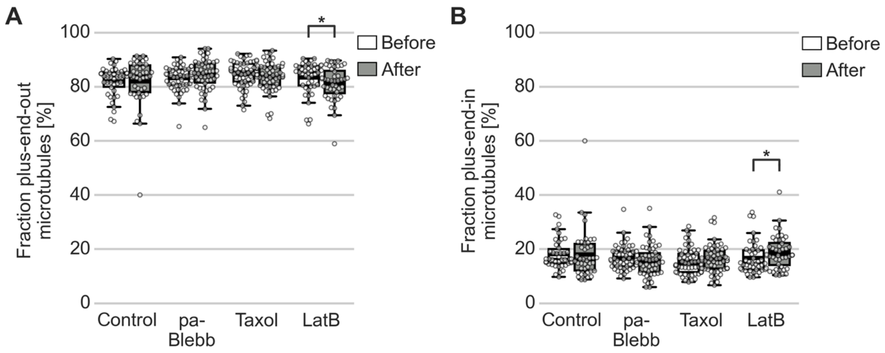

Fig. S15. Treatments that change MT-RF have little effect on MT orientation.

Neurons expressed the microtubule plus tip tracker EB3 fused to the fluorophore mNeonGreen. After one day in culture neurons were imaged for 1 min, then were treated with 6 nM taxol, 40 µM para-amino Blebbistatin (pa-Blebb) or 10 µM latrunculin B (LatB) for 3.5 to 7 hours and neurons were imaged again for 1 min. Microtubule orientation was measured from EB3 comet movement direction relative to the soma. EB3 comets were traced using the plusTipTracker function of u-track and traces automatically processed with a custom-made Python package. (**A** and **B**) Fraction of microtubules (**A**) plus-end-out and (**B**) plus-end-in. (n = 42 cells for control, n = 50 cells for pa-Blebb, n = 55 cells for taxol, n = 44 cells for LatB; N = 3 independent experiments). Thick line in boxplots represents the mean. *P <0.05, Wilcoxon signed-rank test.

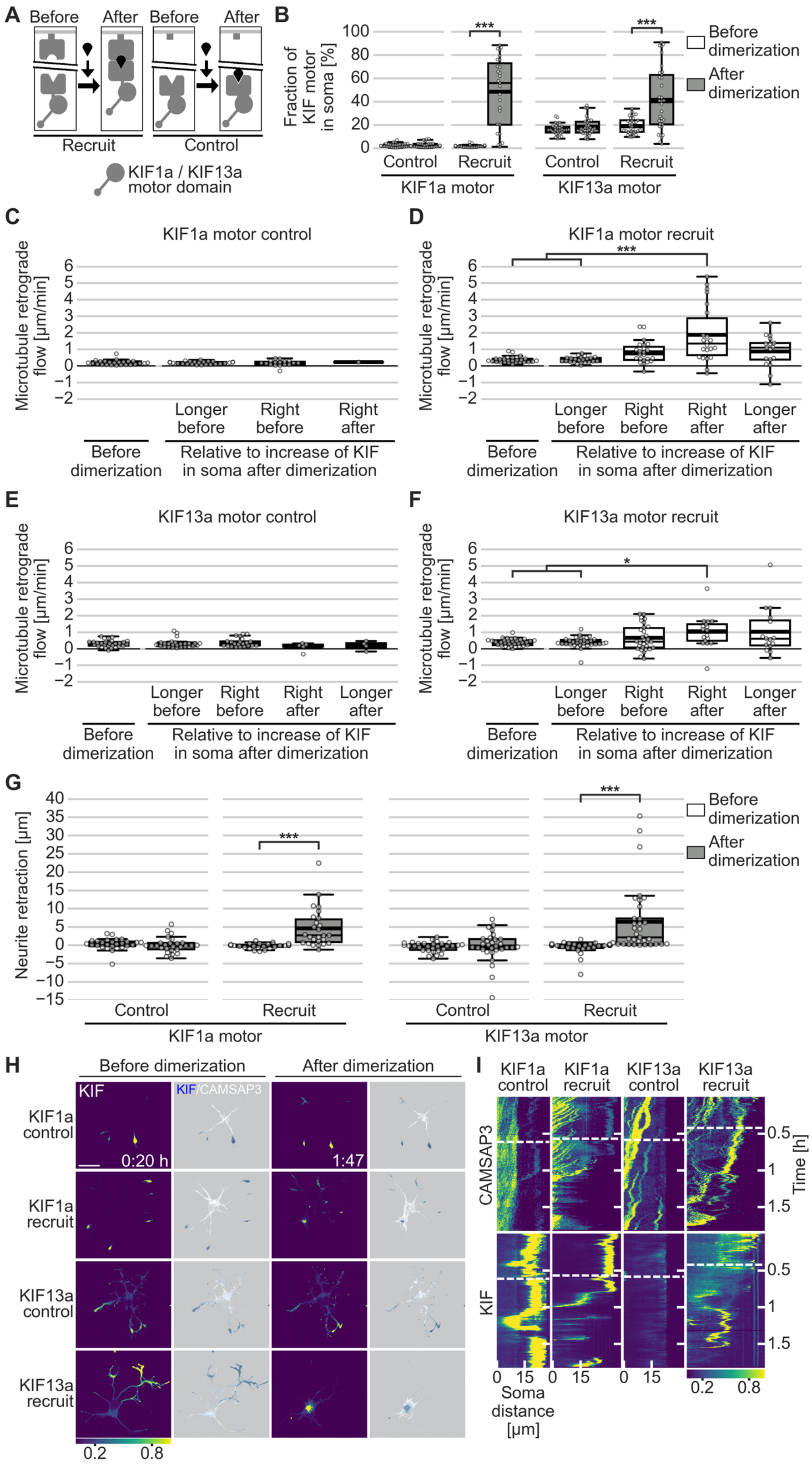
A
Before
After
Before
After
Recruit
Control
KIF1a / KIF13a motor domain
B
Fraction of KIF motor in soma [%]
Before dimerization
After dimerization
Control
Recruit
KIF1a motor
KIF13a motor
C
KIF1a motor control
D
KIF1a motor recruit
E
KIF13a motor control
F
KIF13a motor recruit
Microtubule retrograde flow [µm/min]
Longer before
Right before
Right after
Longer after
Before dimerization
Relative to increase of KIF in soma after dimerization
G
Neurite retraction [µm]
H
Before dimerization
After dimerization
KIF
KIF/CAMSAP3
KIF1a control
KIF1a recruit
KIF13a control
KIF13a recruit
0:20 h
1:47
I
CAMSAP3
KIF
Time [h]
Soma distance [µm]

Fig. S16. Recruiting kinesin motor domains to the membrane speeds up MT-RF and retracts neurites after motor domain movement to the soma.

The motor domains of the kinesins KIF1a (amino acids 1-405) and KIF13a (amino acids 1-639) were recruited to the membrane anchor C2 using the rapalog system. Neurons expressed the fluorophore mNeonGreen fused to CAMSAP3, FRB-C2-Cerulean3 (recruit) or Cerulean3-C2-Cerulean3 (control) and KIF1aMotor-mScarlet-FKBP (KIF1a motor) or KIF13aMotor-mScarlet-FKBP (KIF13a motor). Neurons were imaged after one day in culture for 16 to 60 min, then 62.5 nM of the rapalog A/C dimerizer was added and neurons were imaged further. (**A**) Illustration of the dimerization experiment. (**B**) Fraction of KIF mass in the soma was calculated as the fraction from the KIF mass in the entire neuron. The soma fraction was averaged before dimerization and from 60 to 90 min after dimerization. Soma fraction were compared before and after dimerization. (**C** to **F**) MT-RF was measured from CAMSAP3 traces and averaged in time ranges relative to the timepoint of dimerization and the timepoint with (**C** and **D**) a soma fraction of KIF1a of at least 25% or (**E** and **F**) a soma fraction of KIF13a of at least 50%. MT-RF was averaged before dimerization and for several time ranges after dimerization: more than 7 min before soma fraction increase (longer before), from 0 to 7 min before soma fraction increase (right before), from 0 to 7 min after soma fraction increase (right after) and from 8 to 14 min after soma fraction increase (longer after). If a cell did not reach the defined threshold of soma fraction of the kinesin motor, "longer before" and "right before" were relative to the end of the movie and the cell did not have data for "right after" and "longer after". For [E] no cell had data from "longer after" soma fraction increase. (**G**) Neurite retraction was calculated in relation to the average neurite length of the last 15 min before dimerization and averaged from 60 to 90 min after dimerization. Retraction was compared before and after dimerization. (**H**) Representative neurons showing KIF distribution in the neuron before and after dimerization. Color coded KIF intensity shown (blue lower, yellow higher intensity). Dimerization was done between 00:25 and 00:40 min. Scale bar, 20µm. (**I**) Color coded CAMSAP3 and KIF intensity (blue lower, yellow higher intensity) along one neurite from each neuron in [H] (x-axis) over time (y-axis; kymograph). (for KIF1a control n = 30 for [B], of those n = 25 for [C] and n = 27 cells for [G]; for KIF1a recruit for [B, D and G] n = 27 cells; for KIF13a control for [B] n = 31 cells, of those n = 28 cells for [E] and

n = 30 cells for [G]; for KIF13a recruit n = 34 cells for [B], of those n = 33 cells for [F and G]). Thick line in boxplots represents the mean. ***P <0.001, *P <0.05, Wilcoxon signed-rank test for [B and G] and Kruskal Wallis multiple comparison with Dunn's posthoc test with Bonferroni correction for [C to F].

Supplementary Tables

Table S1. List of primers.

Related to methods, section DNA constructs. Provided as separate file.

Table S2. List of plasmids used for transfections.

Related to methods, section Neuron transfections.

Name	Source	Identifier
pBa-tdTomato-bicDN-FKBP	(60)	Addgene plasmid #64205
pHalo-N1-TUBB5-Halo	(13)	Addgene plasmid #64691
pBetaActin-tandem-mCherry	Sebastian Dupraz	N/A
pBetaActin-EB3-mNeonGreen	Stanislav Vinopal	N/A
pBetaActin-TUBB2a-mEos3.2	This paper	Addgene plasmid #191320
pBetaActin-TUBB2a-Dronpa	This paper	Addgene plasmid #191321
pBetaActin-mNeonGreen-CAMSAP3	This paper	Addgene plasmid #191322
pBetaActin-TUBB2a-mNeonGreen	This paper	Addgene plasmid #191323
pBetaActin-TUBB2a-mScarlet	This paper	Addgene plasmid #191324
pBetaActin-IC2N-dCer3	This paper	Addgene plasmid #191325
pBetaActin-FRB-dCer3-CAAX	This paper	Addgene plasmid #191326
pBetaActin-FRB-C2-dCer3	This paper	Addgene plasmid #191327
pBetaActin-dCer3-C2-dCer3	This paper	Addgene plasmid #191328
pBetaActin-Halo-CAMSAP3	This paper	Addgene plasmid #191329
pBetaActin-FKBP-mScarlet-Dync1h1motor	This paper	Addgene plasmid #191330
pBetaActin-FKBP-mScarlet-Dync1h1motorK2599T	This paper	Addgene plasmid #191331
pBetaActin-FKBP-Dync1h1motor	This paper	Addgene plasmid #191332
pBetaActin-FKBP-Halo-Dync1h1motor	This paper	Addgene plasmid #191333
pBetaActin-FKBP-mScarlet-KIFC1motor	This paper	Addgene plasmid #191334
pBetaActin-FKBP-mScarlet-KIFC1motorN593K	This paper	Addgene plasmid #191335
pBetaActin-KIF1aMotor-mScarlet-FKBP	This paper	Addgene plasmid #191336
pBetaActin-KIF13aMotor-mScarlet-FKBP	This paper	Addgene plasmid #191337
pBetaActin-TUBB2a-mEos3.2-mEos3.2-mEos3.2	This paper	Addgene plasmid #191338

Supplementary Movies

Movie S1. Microtubules move retrogradely towards the soma.

Data from Fig. 1C. Neurons expressing tubulin subtype TUBB2a fused to photoconvertible fluorophore mEos3.2 were cultured for one day and then imaged before axon development. The filled white circle indicates the time of photoconversion and photoactivation at 0:00 min, with white arrows indicating the site of photoconversion. Scale bar, 20 μm.

Movie S2. Microtubules move retrogradely in a synchronous manner.

Data from Fig. 1F. Neurons without an axon expressed the tubulin subtype TUBB2a fused to the photoactivatable fluorophore Dronpa, were cultured for one day and then imaged. The filled white circle indicates the time of photoconversion and photoactivation at 0:00 min, with white arrows indicating the sites of photoconversion. Scale bar, 20 μm.

Movie S3. MT-RF slows down in the axon.

Data from Fig. 2C. The tubulin subtype TUBB2a was fused to the photoconvertible fluorophore mEos3.2, expressed in neurons imaged after one day in culture. The filled white circle indicates the time of photoconversion at 0:00 min with white arrows indicating the sites of photoconversion. Scale bar, 20 μm.

Movie S4. MT-RF is similar in proximal and distal parts of the axon.

Data from fig. S5C. Neurons expressing the tubulin subtype TUBB2a fused to the photoconvertible fluorophore mEos3.2 were imaged after one day in culture. The filled white circle indicates the time of photoconversion at 0:00 min, while white arrows indicate the sites of photoconversion. Scale bar, 20 µm.

Movie S5. MT-RF slows down in axons of neurons cultured in 3D.

Data from Fig. S6C. Neurons expressing tubulin subtype TUBB2a fused to photoconvertible fluorophore mEos3.2 were cultured in 3D collagen matrices and imaged after one day in culture. Maximum intensity projection is shown. The filled white circle indicates the time of photoconversion at 0:00 min with white arrows indicating the sites of photoconversion. Scale bar, 20 µm.

Movie S6. MT-RF slows down in axons of neurons in brain slices.

Data from Fig. 2F. To image neurons in brain slices, embryonic cortices were electroporated *ex utero* with TUBB2a fused to triple mEos3.2, brains were sliced coronally, kept in culture for 2 days and then imaged. Maximum intensity projection is shown. The filled white circle indicates the time of photoconversion at 0:00 min with white arrows indicating the sites of photoconversion. Scale bar, 20 µm.

Movie S7. Later in development MT-RF slows down in dendrites and axons.

Data from fig. S7B. The tubulin subtype TUBB2a was fused to the photoconvertible fluorophore mEos3.2, expressed in neurons and then imaged after 6 to 7 days. The filled white circle indicates the time of photoconversion at 0:00 min with white arrows indicating the sites of photoconversion. Scale bar, 20 µm.

Movie S8. MT-RF slows down shortly after axon outgrowth.

Data from Fig. 3, D and E. The microtubule minus-end binding protein CAMSAP3 was fused to the fluorophore mNeonGreen and expressed together with the cytosolic marker td-mCherry (cytosol). Fusion proteins were expressed in neurons and imaged after one day in culture. The white arrows indicate the timepoint at which the axon grew out. Scale bar, 20 µm.

Movie S9. MT-RF does not continuously slow down before axon formation in neurites with axon-like properties.

Data from Fig. 4D. The marker for axon-like properties caKIF5C was fused to the fluorophore Cerulean3 and expressed together with the tubulin subtype TUBB2a fused to the photoconvertible fluorophore mEos3.2. Neurons without an axon were imaged after one day in culture. White arrows in the photoconverted and non-photoconverted channels indicate the site that was photoconverted at time 0:00. The white arrow in the caKIF5C channel points to neurites with caKIF5C accumulation. Scale bar, 20 µm.

Movie S10. Microtubule density cycles are reduced in the axon.

Data from Fig. 5, C and D. The tubulin subtype TUBB2a was fused to the fluorophore mScarlet and expressed in neurons. Neurons were imaged after one day in culture. The bigger white arrow appears at the time when the axon grows out and points to the neurite that becomes the axon. Tubulin intensity for the two neurites marked with smaller white arrows was calculated by normalizing the average tubulin intensity in the neurite by the tubulin expression level in the neuron, modelled as a linear function of the average intensity in all neurites for each timepoint. Scale bar, 20 µm.

Movie S11. Treatment with compounds that stabilize multiple axon identities slows down MT-RF.

Data from Fig. 6C. The tubulin subtype TUBB2a was fused to the photoconvertible fluorophore mEos3.2 and expressed in neurons. Neurons were cultured for one day and then treated with control (DMSO), 40 μM pa-Blebb or 6 nM taxol for 20 – 240 min and directly imaged. The filled white circle indicates the time of photoconversion at 0:00 min with white arrows indicating the site of photoconversion. Scale bar, 20 μm.

Movie S12. Treatment with pa-Blebb and taxol reduces microtubule density cycles.

Data from Fig. 7, C and D. The tubulin subtype TUBB2a was fused to the fluorophore mNeonGreen and expressed in neurons. After one day in culture, neurons were treated with control (DMSO), 40 μM pa-Blebb or 6 nM taxol for 120 min and then imaged. Tubulin intensity was obtained by normalizing the average tubulin intensity in the neurite (indicated by white arrows) by the tubulin expression level in the neuron, modelled as a linear function of the average intensity in all neurites for each timepoint. Scale bar, 20 μm.

Movie S13. Inhibiting dynein slows down MT-RF.

Data from Fig. 8, D and E. Tubulin subtype TUBB2a was fused to the photoconvertible fluorophore mEos3.2. for chronically inhibiting dynein, I2CN-Cerulean3 was expressed in neurons. Neurons were imaged after one day in culture. For acutely inhibiting dynein, neurons were treated with control (DMSO) or 50 μM Ciliobrevin-A for 30 min directly before imaging. The filled white circle indicates the time of photoconversion with white arrows indicating the site of photoconversion. Scale bar, 20 μm.

Movie S14. Recruiting endogenous dynein to the membrane speeds up MT-RF.

Data from Fig. 9D. mNeonGreen-CAMSAP3, tdTomato-bicDN-FKBP and FRB-Cerulean3-CAAX were expressed in neurons. After one day in culture, neurons were

imaged for 24 min, at 0:00 min the 0.5 µM of the rapalog A/C Dimerizer was added, as indicated by the filled white circle, and imaging continued for one hour. Scale bar, 20 µm.

Movie S15. Dynein motor domain recruitment to the membrane in old neurons speeds up MT-RF and leads to microtubule loss in dendrites but not in axons.

Data from fig. S12D. The dynein domain (dynein1 heavy chain 1 amino acids 1453 to 4644, Dync1h1motor) was recruited to the membrane anchor C2 after photoconversion of microtubule patches in neurons cultured for seven days. Neurons expressed the tubulin subtype TUBB2a fused to the photoconvertible fluorophore mEos3.2, FKBP-Halo-Dync1h1motor and FRB-C2-dCerulean3. Microtubule patches were photoconverted and neurons were imaged, then microtubule patches were photoconverted again, 62.5 nM of the rapalog A/C dimerizer were added and neurons were imaged further. The filled white circle indicates the time at which the dimerizer was added at 0:00 min with white arrows indicating the site of photoconversion. Scale bar, 20 µm.

Movie S16. Recruiting dynein motor domain to the membrane speeds up MT-RF but not recruiting motor deficient dynein motor domain.

Data from neurons shown in Fig. 9G. mNeonGreen-CAMSAP3, FRB-C2-Cerulean3 and FKBP-mScarlet-Dync1h1motor (wildtype) or FKBP-mScarlet-Dync1h1motorK2599T (motor-deficient) were expressed in neurons. After one day in culture, neurons were imaged for around one hour, then at 0:00 min, indicated by the filled white circle, 0.5 µM of the rapalog A/C Dimerizer was added, and imaging continued for one hour. Scale bar, 20 µm.

Movie S17. Recruiting the minus-end directed motor KIFC1 to the membrane speeds up MT-RF.

Data from neuron shown in fig. S13C. The wildtype motor domain of KIFC1 (amino acids 125-673) or a motor deficient KIFC1 motor domain (amino acids 125-673, N593K) was

recruited to the membrane anchor C2. Neurons expressed the fluorophore mNeonGreen fused to CAMSAP3, FKBP-mScarlet-KIFC1 and FRB-C2- dCerulean3 and were imaged after one day in culture. Neurons were imaged for one to two hours, then 0.5 μM of the rapalog A/C dimerizer was added to start the recruitment. The filled white circle indicates the time at which the dimerizer was added at 0:00 min. Scale bar, 20 μm.

Movie S18. Recruiting dynein motor domain to the membrane enhances immediate loss of MT density, increases MT density cycles and induces retraction.

Data from Fig. 10, E and F. Neurons expressed mNeonGreen-CAMSAP3, FKBP-mScarlet-Dync1h1 and FRB-C2-Cerulean3 (speedup) or C2-Cerulean3 (control) and were imaged after one day in culture. After imaging for around 30 min, at 0:00 min 62.5 nM of the rapalog A/C Dimerizer was added, and imaging continued for another 90 min. The filled white circle indicates the time at which the dimerizer was added at 0:00 min. White arrows indicate neurites that retracted shortly after dimerization. Scale bar, 20 μm.

Movie S19. Recruiting the dynein motor domain to the membrane after actin depolymerization increases anterograde microtubule flow.

Data from neuron shown in fig. S14C. The dynein domain (dynein1 heavy chain 1 amino acids 1453 to 4644, Dync1h1motor) was recruited to the membrane anchor C2 after 4 to 8 h treatment with 10 μM latrunculin B. Neurons expressed FKBP-Halo-Dync1h1motor, FRB-C2-dCerulean3 and the fluorophore mNeonGreen fused to CAMSAP3. Neurons were imaged after one day in culture, then 62.5 nM of the rapalog A/C dimerizer was added and imaging was continued. The filled white circle indicates the time at which the dimerizer was added at 0:00 min. Scale bar, 20 μm.

Movie S20. Recruiting the dynein motor domain to the membrane after actin depolymerization increases anterograde microtubule flow and neurite growth.

Data from neurons shown in fig. S14, G and H. The dynein domain (dynein1 heavy chain 1 amino acids 1453 to 4644, Dync1h1motor) was recruited to the membrane anchor C2 after 4 to 8 h treatment with 10 µM latrunculin B. Neurons expressed FKBP-Halo-Dync1h1motor, FRB-C2-dCerulean3 and the tubulin subtype TUBB2a fused to the photoconvertible fluorophore mEos3.2. Neurons were imaged after one day in culture, then 62.5 nM of the rapalog A/C dimerizer were added and imaging was continued. The filled white circle indicates the time at which the dimerizer was added at 0:00 min while white arrows indicate the sites of photoconversion. Scale bar, 20 µm.

Movie S21. Treatments that change MT-RF have little effect on MT orientation.

Data from representative neurons of data in fig. S15. Neurons expressed the microtubule plus tip tracker EB3 fused to the fluorophore mNeonGreen. After one day in culture neurons were imaged for 1 min, then were treated with 6 nM taxol, 40 µM para-amino Blebbistatin (pa-Blebb) or 10 µM latrunculin B (LatB) for 3.5 to 7 hours and neurons were imaged again for 1 min. The filled white circle indicates the time after treatment. Scale bar, 20 µm.

Movie S22. Recruiting kinesin motor domains to the membrane speeds up MT-RF and retracts neurites after motor domain movement to the soma.

Data from neurons shown in fig. S16, H and I. The motor domains of the kinesins KIF1a (amino acids 1-405) and KIF13a (amino acids 1-639) were recruited to the membrane anchor C2 using the rapalog system. Neurons expressed the fluorophore mNeonGreen fused to CAMSAP3, FRB-C2-Cerulean3 (recruit) or Cerulean3-C2-Cerulean3 (control) and KIF1aMotor-mScarlet-FKBP (KIF1a motor) or KIF13aMotor-mScarlet-FKBP (KIF13a motor). Neurons were imaged after one day in culture for 16 to 60 min, then 62.5 nM of the rapalog A/C dimerizer were added and neurons were imaged further. The filled white circle indicates the time at which the dimerizer was added at 0:00 min. Scale bar, 20 µm.
